# Spatially Resolved Panoramic in vivo CRISPR Screen via Perturb-DBiT

**DOI:** 10.21203/rs.3.rs-6481967/v1

**Published:** 2025-05-08

**Authors:** Rong Fan, Alev Baysoy, Xiaolong Tian, Feifei Zhang, Paul Renauer, Zhiliang Bai, Hao Shi, Dingyao Zhang, Haikuo Li, Bo Tao, Mingyu Yang, Archibald Enninful, Fu Gao, Guangchuan Wang, Wanqiu Zhang, Thao Tran, Nathan Patterson, Jie Sheng, Shuozhen Bao, Chuanpeng Dong, Shan Xin, Binfan Chen, Mei Zhong, Sherri Rankin, Cliff Guy, Yan Wang, Jon Connelly, Shondra Pruett-Miller, Daifeng Wang, Mina Xu, Mark Gerstein, Hongbo Chi, Sidi Chen

**Affiliations:** Yale University; Yale University; Yale University; Yale University; Yale University; Yale University; St. Jude Children's Research Hospital; Yale University; Yale University; Yale University School of Medicine; Yale School of Engineering and Applied Science; Yale School of Engineering and Applied Science; Chinese Academy of Sciences Center for Excellence in Molecular Cell Science; Aspect Analytics; Aspect Analytics; Aspect Analytics; University of Wisconsin-Madison; Yale University; Yale University; Yale University; Yale University; Yale University; St. Jude Children's Research Hospital; St. Jude Children's Research Hospital; St. Jude Children's Research Hospital; St. Jude Children's Research Hospital; St Jude Children's Research Hospital; University of Wisconsin-Madison; Yale University; Yale University; St. Jude Children's Research Hospital; Yale University

## Abstract

Spatially resolved in vivo CRISPR screening integrates gene editing with spatial transcriptomics to examine how genetic perturbations alter gene expression within native tissue environments. However, current methods are limited to small perturbation panels and the detection of a narrow subset of protein-coding RNAs. We present Perturb-DBiT, a distinct and versatile approach for the simultaneous co-sequencing of spatial total RNA whole-transcriptome and single-guide RNAs (sgRNAs), base-by-base, on the same tissue section. This method enables unbiased discovery of how genetic perturbations influence RNA regulation, cellular dynamics, and tissue architecture in situ. Applying Perturb-DBiT to a human cancer metastatic colonization model, we mapped large panels of sgRNAs across tumor colonies in consecutive tissue sections alongside their corresponding total RNA transcriptomes. This revealed novel insights into how perturbations affect long non-coding RNA (lncRNA) co-variation, microRNA–mRNA interactions, and global and distinct tRNA alterations in amino acid metabolism linked to tumor migration and growth. By integrating transcriptional pseudotime trajectories, we further uncovered the impact of perturbations on clonal dynamics and cooperation. In an immune-competent syngeneic mouse model, Perturb-DBiT enabled investigation of genetic perturbations within the tumor immune microenvironment, revealing distinct and synergistic effects on immune infiltration and suppression. Perturb-DBiT provides a spatially resolved comprehensive view of how genetic knockouts influence diverse molecular and cellular responses including small and large RNA regulation, tumor proliferation, migration, metastasis, and immune interactions, offering a panoramic perspective on perturbation responses in complex tissues.

CRISPR is as a powerful tool for precise genome editing, enabling genetic manipulation to study functional genomics in a variety of biological systems, including developmental biology^1^, neuroscience^2^, cancer^3^, and immunology research^4^, where it has been demonstrated to uncover the impact on gene expression and elucidate tumor initiation, development, and metastasis mechanisms^5^–^7^. Further, high-content large-scale CRISPR screens^8^ enable the discovery of key genetic targets and have been instrumental in systematically perturbing genes to analyze their effects on cellular phenotypes^9^,^10^. Recent advancements in single-cell multi-omics technologies and pooled single-cell CRISPR screening have revolutionized our understanding from genomics to functional genomics by elucidating how genetic alterations affect transcriptional outputs, influence cellular heterogeneities, and key biological pathways at single-cell level^11^–^17^. For example, Perturb-seq combines single cell RNA-seq and CRISPR based perturbations to perform many functional genomic assays in a pool^11^ and discover cell-type-specific transcriptional programs in development or pathogenesis^18^. CRISPR droplet sequencing (CROP-seq) enables pooled CRISPR screens with single-cell transcriptome resolution using droplet microfluidics^15^, taking genetic screening into the single-cell era. It was further expanded to the genome scale to map the genotype-phenotype landscape, predicting the function of thousands of genes at single cell resolution^19^. Deep learning of the single-cell perturbation atlas can further integrate disparate information sources and enhance our understanding of complex, heterogeneous, and dynamic cellular systems^20^,^21^.

However, a critical gap in understanding the role of functional genetic perturbations *in vivo* was the spatial organization of complex tissues like tumors as well as an inability to explore the mechanisms of gene regulation rather than gene expression profiling. The tumor microenvironment (TME) is a spatially organized ecosystem where cell-to-cell interactions and spatial cues play critical roles in cancer development and progression. Understanding how genetic perturbations influence these interactions and the underlying mechanisms is essential for developing effective therapies. Spatial CRISPR genomics developed by Dhainaut et al.^22^ uses ProCode, an antibody-based method, to detect a set of gRNAs and correlate them with spatial transcriptome from a serial tissue section, uncovering tumor clone-specific transcriptional outputs. Other spatial CRISPR screen technologies, largely based on fluorescence in situ hybridization (FISH) imaging, emerge to address the limitation in spatial context by combining CRISPR-based genetic perturbations with various spatial transcriptomic assays, uncovering some of the unknown drivers of tumor clonality while obtaining information about the spatial architecture of the sample, revealing how specific gene perturbations and their associated phenotypes influence neighboring tissue or cellular regions^23^–^30^. However, these methods have yet to achieve joint mapping of perturbations and transcriptome on the same tissue section, often limited by the size and pre-defined nature of the CRISPR libraries used, and as of today, are unable to provide the information beyond gene expression, such as large and small non-coding RNA and post-transcriptional regulation.

To develop next-generation spatially resolved functional genomics, we introduce “Perturb-DBiT” (Perturbation compatible Deterministic Barcoding in Tissue) for spatially resolved, unbiased, pooled *in vivo* CRISPR screening via spatially co-barcoding total RNA transcriptomes and sgRNAs followed by high-throughput base-by-base next-generation sequencing (NGS) (Fig. 1a). This approach provides a panoramic view of *in vivo* perturbation-induced spatial alterations in molecular and cell biology, including gene expression, RNA regulation, cellular dynamics, and cell-cell interaction all in the same tissue section. Crucially, Perturb-DBiT enables the spatial profiling of diverse RNA species, including large and small non-coding RNAs such as lncRNA, microRNA, tRNA, etc., in a spatial *in vivo* CRISPR screen, unveiling their critical regulatory roles in post-transcriptional control, cellular metabolism and function, and their impact on cellular responses following CRISPR perturbations. Through the preservation of spatial architecture, we provide a method to elucidate in several cancer models how genetic perturbations alter RNA regulation and subsequently influence tumor colonization, expansion, dynamics, and tumor-immune interaction. Our method can be potentially expanded to spatial mapping of genetic variants in other high throughput screen systems^31^ or lineage tracing studies^32^.

## Perturb-DBiT Workflow

**Figure 1:**
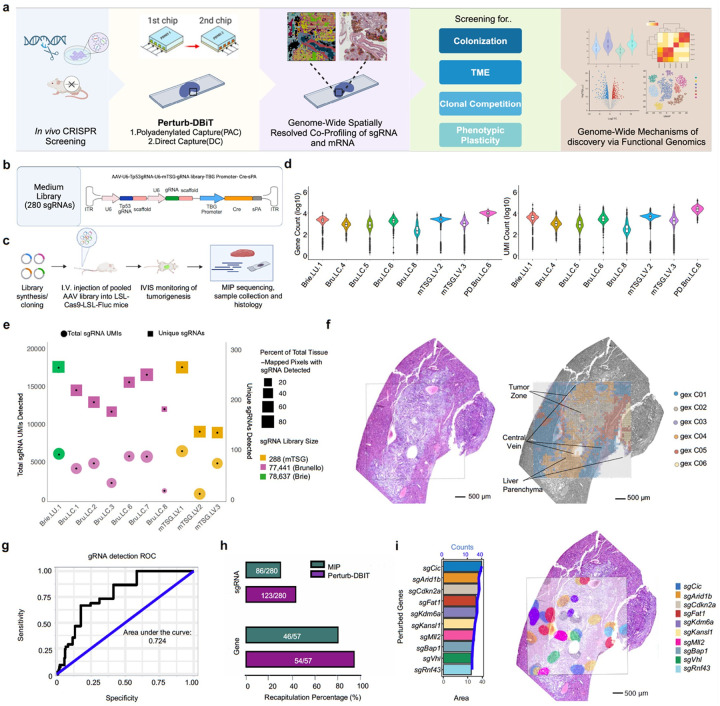
Perturb-DBiT overview, technical performance, and demonstration of robust performance revealing tumor clonal heterogeneity in medium-sized CRISPR-screening libraries. **(a)** Schematic of Perturb-DBiT and its applications. **(b)** Schematic of the AAV-CRISPR vector containing two sgRNA expressing cassettes used for Perturb-DBiT with the medium (288 sgRNA library). **(c)** Schematic of direct *in vivo* AAV-CRISPR liver screen design. **(d)** Violin plot of detected gene/UMI counts per spatial spot from gene expression data across 8 samples utilizing Perturb-DBiT co-profiling. **(e)** Bubble plot depicting Perturb-DBiT sgRNA capture diversity. **(f)** Detection of a medium-sized guide library (288 sgRNAs) in a murine model of autochthonous liver cancer. Left: Liver tissue stained with hematoxylin and eosin (H&E), demonstrating the ROI used for Perturb-DBiT. Right: spatial clustering of gene expression data overlaid on brightfield image, labeled with pathologist annotations. **(g)** Receiver-operator curve comparing the sgRNA detection by 50-micron Perturb-DBiT vs the traditional pooled sequencing method (MIP). (h) Recapitulation analysis comparing sgRNA capture accuracy between Perturb-DBiT and the traditional pooled sequencing method (MIP). **(i)** Left: Bar plot of the top 10 gene perturbations detected by 50-micron Perturb-DBiT. The area and total counts of the gene perturbations are presented by bars and blue line, respectively. Right: Spatial visualization of the top gene perturbations, depicted by color-coded 2D-density maps atop an autochthonous liver tumor ROI.

Perturb-DBiT is developed with two alternative approaches to co-profile single-guide RNA (sgRNA) and spatial transcriptomes within the same CRISPR-screen-mutagenized tissue, which we demonstrate as polyadenylated capture (PAC) and direct-capture (DC) (Extended Data Fig. 1a,b). Both methods are compatible with fresh-frozen (FF) and formalin-fixed paraffin-embedded (FFPE) tissues^33^. In Perturb-DBiT PAC, FFPE CRISPR-screen-mutagenized tissue section is firstly processed with deparaffinization and heat-induced crosslink reversal, while FF tissue processing starts with 1% PFA fixation. Following tissue permeabilization, enzyme-induced *in situ* polyadenylation enables the co-detection of both large and small RNA, as well as sgRNA, by covalently attaching adenosine residues to the 3' end of RNA molecules, forming a poly(A) tail (Extended Data Fig. 1c). Bulk reverse transcription (RT) requires a biotinylated poly-dT reverse transcription primer with a poly(T) oligo, unique molecular identifier (UMI), and a ligation linker (10mer). Next, spatial barcoding is executed using two polydimethylsiloxane (PDMS) microfluidic devices, each featuring 50 or 100 parallel microchannels, and each with channels oriented in directions perpendicular to one another^34^,^35^. Distinct spatial barcodes Ai (i = 1–100, 8mer) and Bj (j = 1–100, 8mer) are utilized with the first and second PDMS device respectively, with imaging after each barcoding step to preserve the tissue's region of interest (ROI) for downstream processing. After lysis and template switching, PCR is used to extract barcoded mRNA cDNA and sgRNA for the downstream workflow. Perturb-DBiT PAC PCR utilizes two primers specific to the TSO-end and Barcode B-end of the transcript. Following PCR, a size selection-based purification approach is then used to achieve separation between endogenous RNA and sgRNA. As the polyadenylation process adds poly(A) tails to all RNAs, and not just the biomolecules of interest, cDNAs originating from rRNAs are selectively removed from amplicons to circumvent the loss of low-abundance transcripts^36^. Separated and purified sgRNA and mRNA cDNA are then used as input for library preparation prior to NGS. Perturb-DBiT DC follows similar tissue preparation (Extended Data Fig. 1d). Following tissue permeabilization, bulk RT is performed using two distinct RT primers: a biotinylated polyd(T) primer and a direct capture primer, each with a UMI and ligation linker (1 or 3, respectively). The polyd(T) primer uses an oligo dT to capture inherently polyadenylated RNA while the direct capture primer uses a “capture” sequence that is complementary to the scaffold sequence on the sgRNA constant region. Here, mRNAs were captured using a barcode Ai (i = 1–50, 8mer), linker 1, barcode Bj (j = 1–100, 8mer), and linker 2, while sgRNAs were captured using a barcode Ci (i = 1–50, 8mer), linker 3, barcode Dj (j = 1–100, 8mer), and linker 4 (see Extended Data Fig. 1d). Through extensive optimization (Extended Data Fig. 2a and Supplementary table S1), we achieved high capture efficiency of the spatial barcodes and sgRNA. After lysis and template switching, Perturb-DBiT DC PCR selectively amplifies sgRNAs and mRNAs using three primers: a common primer at the TSO-end, paired with either a Barcode-D end primer for sgRNAs, or a Barcode B-end primer for mRNAs. The resulting product is then physically separated using SPRI bead purification and used as input for library preparation prior to NGS. Perturb-DBiT PAC and DC offer two complementary methods to co-profile sgRNA and endogenous RNA transcriptome from CRISPR-screen-mutagenized tissue sections while preserving spatial architecture. By combining CRISPR-based genetic perturbations with spatial total RNA transcriptomics, Perturb-DBiT provides a powerful yet flexible tool for unbiased investigation of cell state, function, and dynamics *in vivo* regulated by a variety of RNA molecules in response to genetic alterations, adding a new and integral dimension to spatially resolved multi-omics (Fig. 1a and Extended Data Fig. 1a).

## Perturb-DBiT performance evaluation

We evaluated Perturb-DBiT in several in vivo CRISPR animal models with either FFPE and FF tissues from SMARTA TCR transgenic spleen, B16-OVA mouse tumor, autochthonous mouse liver tumors, HT-29 lung metastatic colonization and E0771 syngeneic lung colonization tumors, representing a broad spectrum of cancer types and cancer immunology experimental designs (Extended Data Fig. 1e). Samples were perturbed with sgRNA pooled screens ranging in size from a small pool of 2 or 86 sgRNAs, to a larger pool of 288 sgRNAs, and extending to the use of genome-scale sgRNA libraries containing approximately 80,000 gRNAs. Furthermore, we successfully employed Perturb-DBiT with lentivirus, retrovirus, and AAV viral vector delivery methods. This versatility underscores Perturb-DBiT's adaptability, making it suitable for a broader range of experimental applications. Perturb-DBiT consistently generated high-quality spatial transcriptomics data across diverse tissue types (Fig. 1d). Notably, one PAC HT-29 lung metastatic colonization FFPE sample yielded an average of 3,371 genes and 6,429 UMIs per spot for exonic molecules, while intronic molecules had a mean of 7,903 genes and 21,821 UMIs per spot. On the other hand, an adjacent DC HT-29 lung metastatic colonization FFPE sample yielded 1,467 UMIs and 1,130 unique genes per spot, suggesting that the PAC method may outperform DC in mRNA capture. These results demonstrate Perturb-DBiT's ability to capture comprehensive total RNA transcriptional profiles, even in challenging FFPE samples, in an untargeted genome-scale manner.

**Figure 2:**
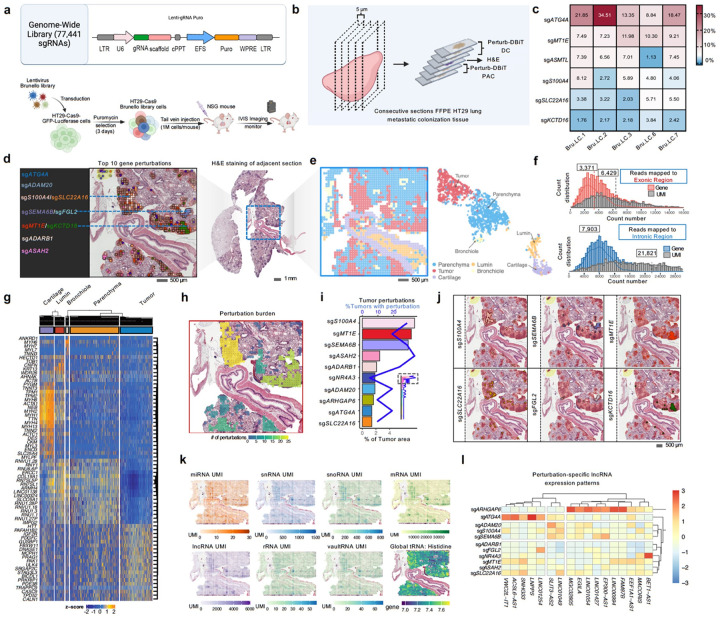
Perturb-DBiT mapping reveals high-resolution tumor clonality by integrating with histology. ( **a)** Top: Schematic of Lenti-gRNA Puro construct. Right: Schematic showing the HT29 lung metastatic colonization model. Bottom: Schematic showing the development of HT29 lung metastatic colonization model. **(b)** Schematic depicting six adjacent sections taken from FFPE HT29 metastatic colonization tissue, three of which were used for Perturb-DBiT DC, two for Perturb-DBiT PAC, and the middle section used for H&E staining. **(c)** Relative abundance of sgRNA-associated UMIs within the Region of Interest (ROI) compared to total UMIs, for the top six sgRNA hits across the six sections shown in 2B. **(d)**Right: Spatial mapping of tissue sections collected from HT29 lung metastatic colonization model transduced with genome-scale Brunello sgRNA library. Left: Combined spatial distribution of top 10 sgRNAs. **(e)** Left: Unsupervised clustering of the combined exonic and intronic expression matrix revealed five transcriptomic clusters. Right: UMAP analysis revealing the five distinct clusters closely matching tissue histology: parenchyma, tumor, cartilage, lumin, and bronchiole. **(f)** Distribution of detected gene/UMI counts per spatial spot from reads mapped to exonic or intronic region. **(g)** Top ranked DEGs defining each cluster in Figure 2E. **(h)** Spatial distribution of perturbation burden calculated as the area of spots with a detected sgRNA within a specified tumor region. **(i)** Bar plot of the top 10 gene perturbations detected by 50-micron Perturb-DBiT. The area and total counts of the gene perturbations are presented by bars and blue line, respectively. **(j)** Spatial colocalization of top enriched sgRNA pairs. **(k)** Spatial count maps of different non-coding RNAs and global tRNA histidine plot. **(l)** Heatmap depicting perturbation-specific lncRNA expression patterns with the following filters: adj. p-val<0.05, absolute-log-fold-change >1, and gene detection difference between groups >25%.

Perturb-DBiT demonstrated robust sgRNA capture performance across a range of CRISPR screen libraries and experimental conditions (Fig. 1e and Supplementary Table S1). We successfully and unbiasedly detected from 2/2 to 265/288 unique sgRNAs in our regions of interest (ROI) and between 754 and 17,548 unique total sgRNA UMIs with 1–106 QC-passed sgRNA UMIs per spot, dependent on the size of the CRISPR screen library and the sample type. Notably, we were able to detect 5,740 unique sgRNA UMIs (1–26 UMIs/spot) from a genome-scale-CRISPR library-mutagenized PAC lung metastatic colonization sample, accounting for all 52% of the tissue-containing spots and representative of 250 unique sgRNAs. As expected, the detected sgRNAs are a fraction of the whole library due to the tissue mapping region representing only a fraction of the whole tumor, yet the result obtained from a single tissue slide is already the among the largest number of perturbations detected in a spatial in vivo CRISPR screen. To assess the consistency of sgRNA capture multiple across sections, we analyzed a series of adjacent sections of genome-scale CRISPR screen-mutagenized HT-29 lung metastatic colonization samples with similar ROIs (Fig. 2c). Between five adjacent sections, we were consistently able to capture the top six sgRNAs hits, demonstrating the ability for Perturb-DBiT to capture the most important and biologically relevant genetic perturbations.

## Perturb-DBiT ensures precise sgRNA fidelity and recapitulation across small and medium sized CRISPR screening libraries

To demonstrate Perturb-DBiT's broad applicability for spatially profiling sgRNAs, we started with three models using small (2 NTC or 86 sgRNA) and medium-sized (288 sgRNA) CRISPR libraries and different modes of CRISPR vector delivery (Fig. 1b and Extended Data Fig. 2a). For the small sgRNA library, B16 (melanoma) OVA-Cas9-GFP cell lines were transduced with retroviral CRISPR sgRNA library targeting 38 epigenetic genes (two sgRNA sequences per gene and 10 NTCs). Perturb-DBiT PAC enabled the efficient capture of 78 out of 86 sgRNAs (< = 35 UMIs/spot), with sgRNAs detected in 90% of all tissue-bearing spots, demonstrating robust spatial and sgRNA coverage. Specificity was demonstrated by significant overlap between sgRNAs targeting the same gene relative to the ROI (Extended Data Fig. 2c). We applied the same dual-guide direct capture lentiviral sgRNA vector with 2-NTCs to an immune-editing model transducing only CD4 + SMARTA T cells^37^ in splenic tissue. Perturb-DBIT DC again revealed overlapping NTC sgRNAs (1–5 sgRNA UMI/spot) relative to the ROI. We further utilized immunofluorescence (IF), revealing sgRNA alignment with the B-cell zone, suggesting that CD4 + SMARTA cells are mostly differentiated to follicular helper (Tfh) cells and interact with B cells at day 6 post-LCMV infection^38^ (Extended Data Fig. 2b). Together, these models demonstrate Perturb-DBiT DC and PAC's efficiency and accuracy for sgRNA capture in tumor and immune cells.

We next applied Perturb-DBiT to profile sgRNAs from FFPE samples derived from previously published CRISPR genetically engineered mouse models (CRISPR-GEMMs) of liver cancer (Fig. 1c), utilizing a mouse tumor suppressor gene (mTSG) CRISPR library targeting the 56 most frequently mutated tumor suppressor genes from pan-cancer datasets within The Cancer Genome Atlas (TCGA)^39^,^40^. The mTSG RNA library was delivered by AAV into LSL-Cas9 knock-in mice^41^, utilizing an all-in-one construct that included both sgRNA and a liver specific promoter (TBG promoter) to drive Cre specific expression in liver cells. AAV-CRISPR-mediated pooled mutagenesis drives autochthonous liver tumorigenesis in this immunocompetent mouse. Following mutagenesis, the mouse received immune checkpoint blockade (ICB) treatment of anti-PD-1 (aPD-1) and samples were processed for Perturb-DBiT DC sgRNA capture. SgRNAs were detected in nearly 75% of the ROI profiled, with up to 106 UMIs detected per spot (Extended Data Fig. 2d,e). Notably, Perturb-DBiT captured 263/288 sgRNAs in the mTSG panel. Since AAV vectors do not integrate into the host cells' genomic DNA, unlike lentiviral vectors in most CRISPR screens, this study used molecular inversion probe sequencing (MIPS) to capture the mutational landscape and test sgRNA activity. Comparatively, the previously published MIPS analysis from multiple sections of the same tissue block detected 86 unique sgRNAs overlapping with our data, validating that Perturb-DBiT recapitulates 100% of the enriched sgRNAs from the MIPS analysis^42^. Moreover, Perturb-DBiT demonstrates remarkable sensitivity, detecting roughly 3-fold the amount of sgRNAs captured by MIPS in just a 1.25×1.25 mm ROI, highlighting the high sensitivity and capture efficiency to detect sgRNA perturbations using spatial Perturb-DBiT as opposed to the gold-standard bulk *in vivo* CRISPR screen analysis.

### Perturb-DBiT enhanced sgRNA capture and transcriptome spatial mapping reveals tumor origin and oncogenic pathways in liver using CRISPR-mediated genetically engineered mouse models (GEMMs)

After confirming successful recapitulation of sgRNAs in the aPD-1-treated liver CRISPR-GEMM sample, we applied Perturb-DBiT PAC co-profiling to an adjacent section of the same treated liver block using a 50-micron spot size device to capture a larger ROI (5×5mm). Perturb-DBiT detected an average of 5,092 UMIs/spot and 2,595 genes/spot and unsupervised clustering revealed 6 distinct transcriptomic clusters characterized by differentially expressed gene (DEG) markers. Spatial clustering aligned closely with the histology from an adjacent section stained with hematoxylin and eosin (H&E), delineating tumor zones, liver parenchyma, and the central vein (Fig. 1f, and Extended Data Fig. 2f,g). H&E analysis revealed carcinoma-like features, indicative of an epithelial origin for the primary tumor. Consistent with this, the analysis of DEGs in tumor spatial clusters gexC02 and gexC03 revealed upregulation of *Epcam* and *Krt19*, markers commonly associated with epithelial cells^43^. The upregulation of *Prom1* further suggested the presence of stem cell-like properties, consistent with its identification as an important liver cancer stem-like cell (CSC) and the role of *Prom1*-derived cells in expanding epithelial tumor cells in human hepatocellular carcinoma (HCC)^44^. Perturb-DBiT PAC again demonstrated superior sgRNA capture efficiency of the mTSG-targeting CRISPR-screen library compared to traditional pooled amplicon sequencing via MIPS. In the same tissue section, Perturb-DBiT captured 43% of total sgRNAs, while MIPS captured only 30%^42^. Additionally, Perturb-DBiT recapitulated 95% of the 57 mTSG target genes, capturing more sgRNA diversity than the gold-standard MIPS (80%) (Fig. 1h). Receiver-operator curve analysis revealed a trend toward improved true sgRNA detection accuracy compared to the published MIPS data with an AUC of 0.724 (Fig. 1g). While an acceptable model for correct sgRNA classification, this recapitulation result is particularly remarkable given the spatial sequencing nature of Perturb-DBIT and the select ROI analyzed compared to the traditional bulk pooled sequencing. We identified the top 10 sgRNA hits using Perturb-DBiT, which exhibited a heterogeneous spatial distribution, reflecting the inherent genetic and phenotypic diversity of primary tumors (Fig. 1i). We next integrated sgRNA and transcriptomics profiling through dimensional reduction of Mixscape perturbation scores (pUMAP), which allowed us to differentiate cell populations based on their response to specific perturbations (Extended Data Fig. 2h). This defined clusters by their major perturbation (> = 20% of clustered spots) and included non-perturbed (NP) spots serving as a reference point to enable a direct comparison between perturbed and NP cells. Notably, top pUMAP cluster *sgMll2*, also emerged as a top depleted hit from the MIPS data yet is enriched in this dataset. We utilized violin plots to analyze oncogene expression between pUMAP clusters, which correspond to distinct functional states within the tissue (Extended Data Fig. 2h). pUMAP cluster sg*Cdkn2a*, a well-known tumor suppressor and senescence gene, is found to impact multiple oncogenes in this analysis, suggesting it as an important player in the network of oncogenic regulation. Further, genetic alterations of *Cdkn2a* have been shown to indicate HCC tumors with poor prognosis, suggesting a role of the p16 pathway in tumor aggressiveness^45^. Differential expression (DE) analysis of each pUMAP cluster using pseudo-replicate Wilcox tests (Extended Data Fig. 2j) revealed significant insights. The *Bap1* pUMAP cluster demonstrated upregulation of sg*Zscan20* – critical for liver carcinoma development – indicating compensatory transcriptional responses to disrupted chromatin dynamics^46^. Gene set enrichment analyses (GSEA) (Extended Data Fig. 2i) further identified key biological pathways, notably in the sg*Cdkn2a* cluster, which is linked to negative regulation of apoptosis. This suggests that sg*Cdkn2a* knockout may allow damaged or mutated cells to survive, contributing to cancer progression, a mechanism that has been seen in colorectal cancer^47^. These results underscore the roles of functional genomics in shaping liver tumor biology and offer potential insights for interventions. Further, the spatial distribution of sgRNAs and transcriptomic profiles might reflect responses to immune checkpoint inhibition by PD-1, which can alter gene expression patterns and TME architecture.

### Perturb-DBiT for in vivo screening of lung metastatic colonization using a genome-scale CRISPR library

Given Perturb-DBiT's ability to perform base-by-base sequencing of sgRNAs for precise identification of genetic perturbations, the method is not inherently limited by the size of the starting library. However, the effective coverage of the screening ultimately depends on sequencing depth and the extent of tissue mapping area. Here we sought to test it with a genome-scale human CRISPR knockout pooled library (Brunello) for *in vivo* screening of tumor cell colonization in the lung through intravenous injection. This library contains 77,441 sgRNAs targeting 19,114 genes, with an additional 1,000 non-targeting controls. We transduced HT-29 cells, a human colorectal cancer cell line, with a pool of Brunello lentiviral vectors encoding the genome-scale CRISPR-Cas9 gene knockout library (Fig. 2a). We used low MOI to maximize the percentage of single integrants in the transduce cell population. Intravenous injection of HT29-Cas9-Brunello library cells into 8-week-old NSG mice led to *in vivo* tumor colony formation^48^. After 35 days, when tumor colony size is moderate as detected by IVIS, lung tissues were collected for Perturb-DBiT PAC using a 50-micron device. Within the ROI, we identified 235 unique sgRNAs and a total of 5,740 sgRNA UMIs. To assess reproducibility and coverage, we analyzed five adjacent sections of CRISPR screen-mutagenized HT-29 lung metastatic colonization samples with similar ROIs (Fig. 2b,c). Figure 2c highlights the percentage of sgRNA UMIs detected within the ROI relative to all sgRNA-associated UMIs. Notably, although it was not demonstrated or perhaps not always necessary to detect all sgRNAs in this genome-scale library, our results revealed consistent enrichment of specific sgRNAs, including sg*ATG4A*, sg*MT1E*, and sg*S100A4*, across multiple tissue sections, demonstrating the capture of the functionally most relevant perturbations in this model driving metastatic colonization.

We mapped all top sgRNA hits onto the H&E image using a platform provided by Aspect Analytics (Genk, Belgium). Top 10 sgRNA hits were highlighted and were located within the tumor colonies (Fig. 2d,j and Extended Data Fig. 3A). Perturbation burden varied between tumor colonies, with some regions exhibiting high perturbation density (Fig. 2h), suggesting differential selection pressures within distinct tumor areas. Additionally, tumor perturbation frequency varied across genes with sg*S100A4* and sg*MT1E* being the most enriched in this screen (Fig. 2i), indicating their potential role in metastatic tumor adaptation. Interestingly, six of the ten most enriched sgRNAs showed clustering within tumor colonies with instances of colocalization between specific sgRNA pairs, including (sg*S100A4*/sg*SLC22A16*; sg*SEMA6B*/sg*FGL2*; sg*MT1E*/sg*KCTD16*) (Fig. 2d,j). The spatial colocalization of these sgRNAs suggest that they may confer a selective advantage to the tumor cells in which they are expressed and a possible synergistic interaction or clonal cooperation among these genes in the same tumors.

## Perturbation-induced alteration of a variety of coding and non-coding RNA molecules in the tumor colonies

**Figure 3:**
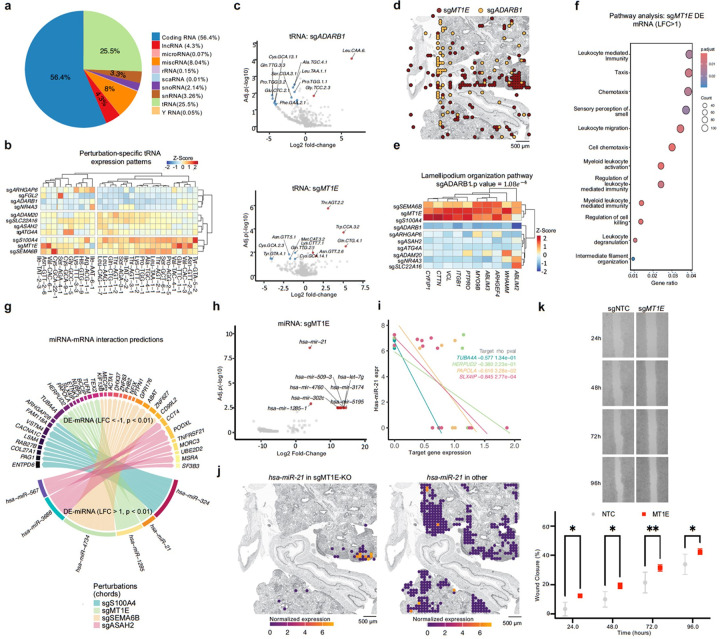
Spatial mapping of small non-coding RNAs in HT29 lung metastatic colonization model. **(a)** Proportion of reads mapped to different RNA categories. **(b)** Heatmap depicting perturbation-specific tRNA expression patterns with the following filters: adj. p-val<0.05, absolute-log-fold-change >1, and gene detection difference between groups >25%. **(c)** Top: DE tRNA volcano plot of sg*ADARB1* perturbation versus all other perturbed spots. Bottom: DE tRNA volcano plot of sg*MT1E*perturbation versus all other perturbed spots. **(d)** Spatial distribution of sg*ADARB1* (yellow) and sg*MT1E* (red) perturbation. **(e)** Heatmap of lamellipodium organization pathway gene expression across top sgRNA perturbations. **(F)** GO Pathway analysis of sg*MT1E* differentially expressed mRNA (LFC>1, adj.p-val>0.05). **(g)** miRNA-mRNA interaction predictions for top sgRNA hits. **(h)** DE miRNA volcano plot of sg*MT1E*perturbation versus all other perturbed spots. **(i)** Inverse correlation between hsa-miR-21 and target gene expression for select target genes from 3I.**(j)** Left: spatial plot of hsa-miR-21(orange) in sgMT1E knockout cells (purple) overlaid on brightfield image of the ROI. Right: spatial plot of hsa-miR-21(orange) in tumor cluster from 2E (purple) overlaid on brightfield image of the ROI. **(k)** Top: Representative images of scratch assay depicting wound closure in HT29 cells with NTC or sg*MT1E* knockdown at 24,48,72 or 96 hours. Bottom: Comparative analysis of wound closure in NTC or sg*MT1E* knockdown cells using a wound healing assay. Statistical significance was determined using two-way ANOVA.

We next evaluated the total RNA capture efficiency of Perturb-DBiT on this lung section. While reads mapped to exonic regions yielded an average of 3,371 genes and 6,429 UMIs per spot, Perturb-DBiT PAC detected a substantially higher number of intronic molecules, likely attributed to the polyadenylation process, resulting in a mean of 7,903 genes and 21,821 UMIs per spot (Fig. 2f), which to our best knowledge is among the highest possible data quality for spatial transcriptome sequencing albeit the FFPE tissue being used. By combining both exonic and intronic gene data while preserving their distinct identities, unsupervised clustering analysis revealed five clusters, each characterized by a unique expression profile and exhibiting strong concordance with the histology (Fig. 2e,g). The spatial distribution of these clusters closely aligned with the histological structures observed in H&E staining. FFPE tissue Perturb-DBiT PAC enabled the capture of diverse non-coding RNA species, revealing robust capture of microRNA, small nucleolar RNA (snoRNA), small nuclear RNA (snRNA), long non-coding RNA (lncRNA), ribosomal RNA (rRNA), and vault RNA, alongside ~ 56% of reads mapping to protein-coding RNA (Fig. 3a). Spatial patterns of these non-coding RNAs demonstrated global enrichment in the tumor regions (Fig. 2k), mirroring the distribution trend observed for mRNAs and sgRNAs. We examined the spatial expression of tRNA isotypes by grouping reads encoding the same amino acids, revealing histidine tRNA globally upregulated in the tumor colony regions of the ROI (Fig. 2k). These findings suggest a potential role for histidine in tumor adaption which aligns with published data demonstrating a link between histidine catabolism and methotrexate sensitivity^49^. Perturb-DBiT uncovered lncRNA expression patterns associated with top sgRNA hits from the CRISPR screen, and subsequent clustering analysis revealed distinct, sgRNA-specific lncRNA covariation patterns, suggesting that these top hits may function as key nodes in lncRNA regulatory networks (Fig. 2l).

To enhance spatial RNA mapping to a near-cellular level while maintaining tissue coverage area, we employed a 100×100 microfluidic device with 20-micron spot size to spatially barcode 100,000 spots on an adjacent lung section (Extended Data Fig. 3b). Clustering analysis of the combined exonic and intronic expression matrix identified seven clusters that exhibited strong concordance with the histology (Extended Data Fig. 3e,f). The spatial distribution of these clusters revealed distinct patterns, highlighting Perturb-DBiT's ability to accurately map fine tissue structures with improved spatial resolution (Extended Data Fig. 3g). Particularly, the tumor cluster closely mirrored the distribution of malignant regions, as evidenced by its strong alignment with H&E staining patterns of the tumor zones (Extended Data Fig. 3h). Examining the top DEGs defining this cluster, *MALAT1* emerged as a key regulator of lung cancer metastasis^50^, while *PTK2* was identified as a promoter of metastasis through its role in regulating tumor cell motility and invasion^51^. Additionally, *NEAT1* was linked to tumor cell metabolism^52^, and *PVT1* was found to function as an oncogene involved in the TGF-β signaling pathway^53^. Notably, *MALAT1*, *NEAT1*, and *PVT1* are non-coding RNAs. Perturbation burden mapping once again revealed that the frequency of tumor perturbations varied across genes, this time with sg*ATG4A* and sg*UBE2U* being those most prevalent (Extended Data Fig. 3c,d).

Recognizing the importance of high-resolution histological images for identifying spatial patterns and leveraging recent advances in machine learning for spatial omics data integration, we applied iStar^54^, a computational framework, to fuse Perturb-DBiT data with H&E histology images. Using the top 3,000 DEGs as input, this approach generated a super-resolved, single-cell transcriptomic map, classifying lung tumor cells into clusters G6, G11, and G13 based on their cellular heterogeneity (Extended Data Fig. 3i). Next, we aimed to perform this imputation analysis using the sgRNA expression profile to link the distribution of sgRNAs with tumor colonies. However, the spatial structure of tumor cells was not clearly revealed using the total number of sgRNAs as input (Extended Data Fig. 3j). Given the high accuracy of top enriched sgRNAs in identifying tumor colonies (Fig. 2d), we selected 15 sgRNAs – present in the majority of sgRNA-containing spots (77.6%) – along with a non-targeting control sgRNA, as the input matrix for the iStar analysis. This analysis spatially resolved 20 clusters, with most sgRNAs exhibiting cluster-specific imputed expression (Extended Data Fig. 3k). For instance, cluster C0 showed enriched expression of sg*UBE2U*, while cluster C16 had notably high expression of sg*DUSP15*. The distribution of these clusters strongly overlapped with tissue structures, as clusters C10 and C14 predominantly overlapping with tumor cell regions. These findings suggest that the combination of Perturb-DBiT and iStar effectively reveal tumor clonal patterns through both gene and top sgRNA expression profiles.

## Spatial profiling of all tRNAs in response to top ranked perturbations in the tumor colonies

**Figure 4:**
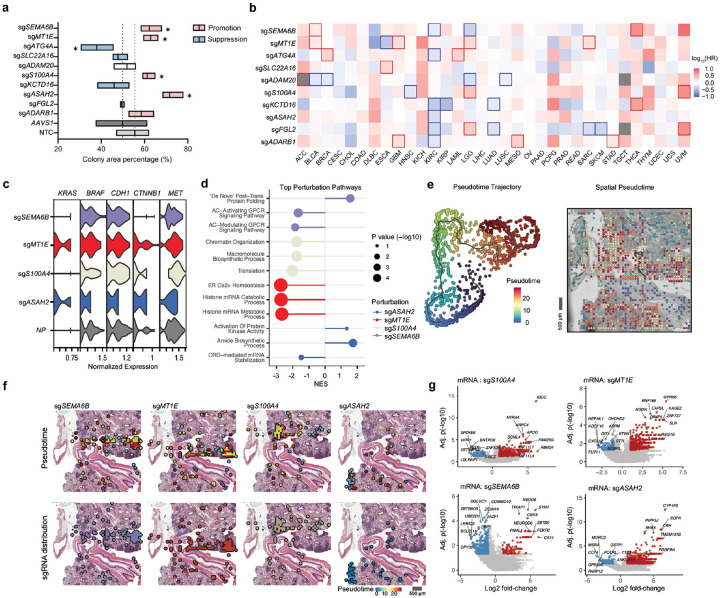
Perturb-DBiT reveals distinct tumor suppressor/promoter programs. **(a)** Colony formation assay results of top enriched sgRNA from HT29 lung metastatic colonization model. **(b)** Hazard Ratio plot of top enriched sgRNA from HT29 lung metastatic colonization model. Squares surrounded by colored boxes represent significant differences in hazard ratios. **(c)** Violin plot of the expression of 5 selected oncogenes, compared between pUMAP clusters. **(d)** Pathway analysis of top perturbations. **(e)** Left: Monocle3 pseudotime analysis of sgRNA detected from HT29 lung metastatic colonization model. Right: spatial pseudotime visualization. **(f)** Top: Pseudotime analysis of top 5 enriched sgRNAs detected by Perturb-DBiT. Bottom: spatial mapping of top 5 enriched sgRNAs detected by Perturb-DBiT, overlaid on the brightfield image of the ROI. **(g)** Volcano plots of the transcriptomic DEG analysis for top enriched sgRNA perturbations.

Perturb-DBiT profiling of non-coding RNAs provides an unprecedented opportunity to uncover the regulatory impact of genetic perturbations on small RNA dynamics. We leveraged Perturb-DBiT PAC to systematically dissect small non-coding RNA responses across top sgRNA hits in the HT-29 lung metastatic colonization model, with a particular focus on the microRNA and tRNA subpopulations, which comprises ~ 1% and ~ 26% of the total mapped RNA, respectively (Fig. 3a). Hierarchical clustering of tRNA expression across top sgRNA hits (Fig. 3b) revealed both sgRNA-specific signatures on tRNAs and distinct global perturbation patterns, stratifying them into three groups based on their impact on tRNAs, highlighting the diversity of tRNA modulation across perturbations. Notably, we observed that sg*FGL2* led to a marked upregulation of serine tRNA expression, suggesting a potential metabolic shift associated with FGL2 loss. *FGL2* is a serine protease with prothrombinase activity, its role in tumor progression extends beyond coagulation to include immune suppression and metabolic regulation^55^. *FGL2* promotes immune evasion by downregulating antigen presentation, inducing B cell apoptosis and suppressing CD8 + T cell activation amongst other effects^56^ and the observed changes in serine tRNA following FGL2 knockout could be linked to alterations in the TME. Our data also revealed that sg*S100A4* knockout distinctly upregulates tyrosine tRNA expression. This finding aligns with previous studies suggesting that *S100A4* expression in cancer is regulated through receptor tyrosine-protein kinase *ERBB2*, which activates the ERK signaling pathway^57^. The upregulation of tyrosine tRNA in the absence of *S100A4* may therefore reflect an adaptive response to this altered signaling, highlighting a potential link between *S100A4* mediated signaling and metabolic shifts in cancer cells. Further, differential tRNA expression analysis for each top sgRNA hit of our screen (Extended Data Fig. 4) revealed that sg*ADARB1* knockout resulted in a global downregulation of tRNAs (Fig. 3c). Given the critical role of tRNAs in protein synthesis, this suggests a profound disruption of protein metabolism, potentially impacting cellular processes reliant on rapid protein turnover, such as cell migration. ADARB1, an A-to-I editing gene, is known to modify tRNAs and other RNA species, influencing their stability and function^58^. Therefore, the observed downregulation of tRNAs could reflect a disruption in *ADARB1*-mediated RNA editing, further exacerbating the metabolic and functional effects in the knockout cells. Spatial mapping of sg*ADARB1* knockout cells (Fig. 3d) revealed their failure to integrate into tumor colonies, instead remaining dispersed in the interstitial space – an outcome distinct from other top ranked perturbations in the screen like sg*MT1E* (Fig. 3c,d), which exhibited global tRNA upregulation and robust tumor colony formation. Given this pronounced migratory deficit and inability to extravasate albeit high viability to survive in circulation, we next examined the key features associated with cell migration such as lamellipodium organization (Fig. 3e), where sg*ADARB1* knockout significantly downregulated genes within this pathway, reinforcing its role in mediating cell motility. To contextualize the specificity of *ADARB1’s* role in migration, we performed pathway-level clustering of DEGs (Extended Data Fig. 5), which revealed a significant effect of *sgADARB1* downregulating genes related to chromatin organization, DNA repair and cell cycle regulation compared to other top perturbations. This finding underscores the essential role of *ADARB1* in maintaining migratory capacity. Conversely, knockouts of *sgSEMA6B, sgMT1E*, and *sgS100A4* resulted in strong upregulation of these lamellipodium-related genes, suggesting a potential inhibitory effect on lamellipodia formation, cell migration, and potentially metastasis (Fig. 3e). *SgSEMA6B* knockout cells led to an upregulation of cysteine tRNAs (Fig. 3b) which are required for filopodia formation^59^. Together with lamellipodia, these related structures contribute to cell motility^60^. This may suggest a potential mechanism by which *sgSEMA6B* regulates cell migration and invasion. Notably, the role of *MT1E* in regulating cell migration has been highlighted in HCC, where knockout of *MT1E* in HCCLM3 cells significantly increased migration^61^. Additionally, xenograft mouse models revealed *MT1E* as a novel tumor suppressor, inhibiting migration and invasion and inducing apoptosis. In our study, we similarly identified *MT1E* as a tumor suppressor (Fig. 4a), further supporting its role in regulating cell motility, migration, and cancer metastasis. To further explore the role of *MT1E* in regulating cell migration, we performed a pathway analysis of *sgMT1E* DE mRNA, which highlighted key pathways such as leukocyte migration, taxis, and cell chemotaxis (Fig. 3f). While the functional implications of these pathways in our model remain to be fully understood, these findings suggest that *MT1E* may influence signaling networks involved in cell movement within the TME.

### Spatial profiling of microRNAs and microRNA-mRNA regulation in responses to top ranked perturbations in the tumor colonies

Beyond tRNA dysregulation, we also observed broad alterations in microRNA (miRNA) expression. Our Perturb-DBiT screen revealed significant changes in several miRNAs (fig. S4), including sg*MT1E* inducing upregulation of multiple microRNAs, notably *miR-21* (Fig. 3h), a well-known oncogenic miRNA that targets numerous tumor suppressor genes associated with proliferation and invasion^62^. Subsequent miRNA-mRNA interaction predictions (Fig. 3g) highlighted putative target genes impacted by sg*MT1E* perturbation, implicating this factor in broader post-transcriptional regulation (Fig. 3i). Strikingly, spatial mapping demonstrated a precise overlay between sg*MT1E* and elevated miR-21 expression (Fig. 3j), suggesting that sg*MT1E* directly or indirectly suppresses *miR-21* expression. MT1E is a key regulator of oxidative stress^63^, and its loss may allow for *miR-21* to escape suppression, promoting tumor progression. Target correlation analysis between mRNA and miRNA revealed that miR-21 may lead to suppression of target genes like *TUBA4A* (Fig. 3g,i), disrupting microtubule dynamics and promoting chromosomal instability, which has been linked to tumor recurrence and metastasis^64^. These findings, together with the striking spatial congruence observed between sg*MT1E* and *miR-21* upregulation (Fig. 3j), underscore a spatially coordinated *MT1E-miR-21* regulatory axis with profound implications for tumor progression and therapeutic targeting. Given the migratory and proliferative effects of *sgMT1E* revealed through both tRNA and miRNA analysis, we validated this transcriptional phenotype in an *in vitro* wound-healing assay (Fig. 3k), which showed a significant increase in wound closure in sg*MT1E* knockdown cells compared to the control, providing functional evidence that *MT1E* inhibits cell migration and proliferation, further implicating its function as a tumor suppressor.

### Spatial in vivo CRISPR screen yields a wide range of mutant colonies recapitulating the tumor differentiation trajectory and dynamics

To evaluate the functional role of the top sgRNA hits detected by Perturb-DBiT in tumor colony formation, we conducted a clonogenic assay of HT-29 cells in vitro (Extended Data Fig. 6a). Briefly, HT-29-Cas9 cells were transduced with either a non-targeting control, *AAVS1* sgRNA control, or target sgRNA lentivirus. *AAVS1* is a well-characterized “safe harbor” locus in the human genome, unlikely to disrupt essential genes if edited and is a reliable positive control^65^. After 3-day selection via puromycin and T7E1 assay validation (Extended Data Fig. 6b) of sgRNA genome editing, tumor cells were seeded at a density of 5,000 cells and resulting cell colonies were analyzed after two weeks. Colony formation assays validated tumor suppressor function of sg*SEMA6B*, sg*MT1E*, sg*S100A*, and sg*ASAH2* from the Perturb-DBiT top ten enriched hits (Fig. 4a and Extended Data Fig. 6c).This was exemplified by the inclusion of four previously reported tumor suppressors (*ARHGAP6*, *CREBZF*, *DHRS7C*, and *NR4A3*)^66^–^69^, whose corresponding sgRNAs were abundantly detected by Perturb-DBiT. Knockout of these genes, except for *DHRS7C*, which was shown to promote tumor colony growth (Extended Data Fig. 6c,d). We next calculated hazard ratios of gene perturbations across various cancer types depicting potential tumor suppressor and promotor activity (Fig. 4b). Notably, in kidney renal clear cell carcinoma (KIRC), most of the top ten sgRNA hits are classified as tumor suppressors, suggesting the target genes' role in growth inhibition. In contrast, other cancers including HT-29, which is classified as colorectal adenocarcinoma (COAD), exhibit a mix of tumor-suppressing and tumor-promoting activities in our data. These results suggest the unique capability of Perturb-DBiT in identifying functional perturbations in a spatially relevant context across this genome-scale sgRNA library.

We next sought to investigate the effect of CRISPR perturbations on the tumor cell transcriptional state and dynamics. First, we examined the relationships between these top perturbations and key oncogenic pathways. We compared the effects of our top four perturbations including NP (non-perturbed) spots serving as a control on genes associated with known HT-29 oncogenic modulators: *KRAS*, *BRAF*, *CDH1*, *CYNNB1*, and *MET* (Fig. 4c). This analysis revealed the diverse functional consequences of the top hits identified by Perturb-DBiT across various oncogenic drivers. Moreover, these results suggest that the top sgRNA perturbations not only vary in their overall effect on cancer types, but also show specificity in how they interact with distinct oncogenic pathways, highlighting Perturb-DBiT's application to elucidating potential pathway-specific vulnerabilities that could be therapeutically targeted. Secondly, we observed that the spatial distribution of these top sgRNA hits typically follows a pattern of both dispersion and concentration within different tumor colonies (Fig. 2j). This suggests that sgRNA enrichment might be indicative of the direction of tumor differentiation or progression. Consequently, by using Monacle3^70^, we conducted pseudotime analysis with spatial transcriptomics data from tumor cells containing sgRNAs. Intriguingly, the distribution patterns of the sgRNAs for sg*SEMA6B*, sg*MT1E*, and sg*S100A4* - three of the four validated tumor suppressors- align well with the tumor differentiation pseudotime trajectory, suggesting the formation of diverse tumor genotypes in a single pooled screen that can recapitulate a spectrum of tumor differentiation dynamics, from regions with fewer sgRNAs to colonies with substantial sgRNA enrichment (Fig. 4e,f). Thirdly, Perturb-DBiT co-profiling data enabled identification of perturbation-associated molecular signatures along the differentiation trajectory. DEG analysis was conducted between spots containing sgRNAs for four tumor suppressors validated in this study and spots containing all other sgRNAs. This revealed significant downregulation (Padj < 0.05, Log_2_FoldChange<-1) of multiple genes known to inhibit tumor growth, along with upregulation (Padj < 0.05, Log_2_FoldChange > 1) of a few oncogenes (Fig. 4g). This may partially explain why knocking out sg*SEMA6B*, sg*MT1E*, sg*S100A4*, or sg*ASAH2* may significantly promote tumor colony growth. Further, Gene Set Enrichment Analysis (GSEA) (Fig. 4d) of the DEGs revealed that knocking out sg*ASAH2* was associated with activation of pathways involved in cellular signaling and regulation, such as protein kinase activity^71^, which plays a role in tumor cell survival, adhesion, and metastatic potential. These findings suggest a potential role for *ASAH2* in modulating key signaling networks involved in tumor progression. In summary, Perturb-DBiT enables efficient *in situ* co-profiling of sgRNAs from a genome-scale human CRISPR library alongside whole transcriptomes. Analysis of the sgRNA data allows for a precise visualization of tumor colonies and rapid identification of functional perturbations on transcriptional state. Concurrently, the transcriptome data analysis reveals genetic modulators that are highly relevant to diverse aspects of tumor biology. For the first time, the integrated analysis of both datasets permits the un-biased spatial uncovering of perturbation-associated molecular signatures including both mRNA and large and small non-coding RNA using a genome-scale CRISPR library.

### Perturb-DBiT identifies genes modulating the tumor architecture and immune composition of the tumor microenvironment in an immune-competent animal model

**Figure 5:**
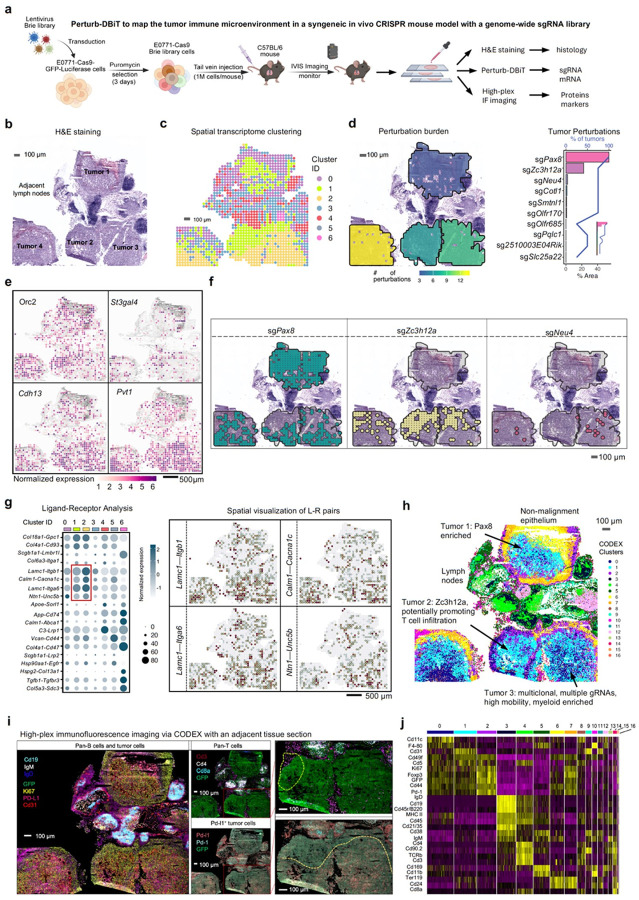
Perturb-DBiT with a CRISPR library in syngeneic metastatic tumor model highlights genes that modulate the structural features of tumors and TME. **(a)** Schematic showing the E0771 syngeneic *in vivo* CRISPR mouse model. **(b)** H&E-stained image of E0771 syngeneic lung fresh-frozen tissue section with labeling of four anatomically distinct tumor regions and adjacent lymph nodes. **(c)** Unsupervised clustering of the gene expression matrix revealing 6 distinct clusters overlain on brightfield image of tissue section (right). **(d)** Left: Spatial distribution of perturbation burden calculated as the area of spots with a detected sgRNA within a specified region. Right: Bar plot of the top 10 gene perturbations detected by 50-micron Perturb-DBiT. The area and total counts of the gene perturbations are presented by bars and blue line, respectively. **(e)** Spatial intensity maps of four selected genes *Orc2, Cdh13, S13gal4, Pvt1* overlain on brightfield image of tissues section. **(f)** Spatial intensity plot of top three sgRNA hits revealed by Perturb-DBiT overlain on H&E image of tissue section. **(g)** Left: ligand-receptor interactions within each of the 6 clusters elucidated by unsupervised clustering of gene expression data. Right: spatial maps of four significant ligand-receptor interaction analyses. **(h)** CODEX staining (26 marker panel) is performed on the adjacent tissue section. Left: Pan B cell markers (Cd19, IgM, and IgD), tumor-specific markers (GFP, Ki67, and PD-L1) and vasculature (Cd31). Middle panel, top: background of GFP-positive (tumor) cells and T cells (Cd3+, Cd4+ helper T cells and Cd3+ Cd8a+ cytotoxic T cells). Middle panel, bottom: Tumor cells showing positivity for Pd-l1. Top right: zoomed in image with green circle representing infiltrating region of Cd8+ T-cells and the tumor. Bottom right: zoomed in image with yellow line representing the border of spatial transcriptomics cluster 1 and cluster 2 within the tumor region. **(i)** UMAP clustering of the CODEX data revealed 17 distinct protein clusters. **(j)** Heatmap showing the top differentially expressed proteins for each cluster.

We next aimed to use Perturb-DBiT to investigate how different gene perturbations impact the TME. To do this, we transduced E0771 cells, a well-characterized mouse syngeneic cell line, with a pool of lentiviral vectors encoding the genome-scale CRISPR-Cas9 gene knockout library (Brie)^48^. The Brie library comprises a total of 19,674 genes and 78,637 unique sgRNAs. Intravenous injection of E0771-Cas9-GFP-Luciferase Brie library cells into 8-week-old C57BL/6 mice led to tumor colony formation (Fig. 5a). After 21 days, lung tissues were collected for Perturb-DBiT PAC co-profiling. Within the ROI (Fig. 5b) containing at least four anatomically identifiable major tumors, a total of 91 unique sgRNAs and 17,548 sgRNA UMIs were identified (Supplementary Table 1 and Extended Data Fig. 7a). Gene ontology (GO) analysis of all sgRNA-targeted genes reveals primary involvement in the regulation of cell growth and developmental processes, highlighting significant roles in stem cell proliferation, cell cycle progression, and various embryonic and tissue development stages (Extended Data Fig. 7b). Concurrently, Perturb-DBiT detected an average of 1,995 genes and 4,458 UMIs per spot (Fig. 1d). Cell type-specific marker genes were then identified by unsupervised clustering, uniquely characterizing their expression within each individual cluster for clear distinction from other groups (Fig. 5c and Extended Data Fig. 7c). Notably, the distribution of these clusters displayed a strong concordance with tissue morphology (Extended Data Fig. 7d). For example, cluster 4 defined lymph nodes (Fig. 5b,c), and cluster 0 almost completely overlaps with airway epithelial cells (Extended Data Fig. 7d). Moreover, we observed that two clusters, cluster 1 (C1) and cluster 2 (C2), exhibited conspicuous and distinctive spatial patterns despite their proximity within the same tumor region (Fig. 5b,c). C1 is defined by DEGs such as *Orc2*, which drives DNA replication^72^ and *St3gal4*, which plays a significant role in tumor progression by promoting neutrophil adhesion to selectins^73^. C2 exhibited upregulation of oncogenes *Pvt*^74^ and *Gse1*^75^, as well as tumor suppressor gene *Cdh13*^76^ (Fig. 5e, and Extended Data Fig. 7e). However, these distinct genetic profiles alone are not sufficient to definitively differentiate the two tumor clusters.

In this instance, we attempt to uncover the mechanisms underlying the distinct characteristics of the two tumor clusters by examining gene perturbations. We selected the top ranked sgRNAs to visualize their spatial perturbation burden and distribution (Fig. 5d,f). Interestingly, we observed a significant enrichment of the sgRNA for sg*Zc3h12a* within Cluster C1. *Zc3h12a* has been established as a key negative regulator of inflammation, and its deficiency leads to a pro-inflammatory environment^77^, which is a driver of immune cell infiltration^78^. Consistent with the perturbed *sgZc3h12a* spatial region in our data, this suggests a localized area of immune dysregulation in the subcluster of C1 proximal to lymph nodes. On the other hand, sgRNA profiles within C2 exhibit marked heterogeneity. NEU4, a sialidase that removes sialic acids from glycoconjugates, has been shown to reduce the motility of hepatocellular carcinoma cells by cleaving sialic acids on CD44, thereby enhancing cell adhesion to the extracellular matrix^79^. Consequently, the preferential enrichment of sg*Neu4* sgRNA in C2 suggests potential mechanisms contributing to increased cellular motility and elevated levels of tumor metastasis. Furthermore, we observed that sg*Pax8* is scarce in C1 but shows a strong distribution pattern within C2 and overlaps with C0 and C1 in Tumor 1 (Fig. 5b,c,f). Functionally, *Pax8* is a lineage-defining transcription factor essential for the survival of differentiated epithelial cells (via *Tp53inp1* regulation)^80^ and its presence across distinct transcriptional clusters indicates that this key regulator is being targeted in various cellular states within this tumor microenvironment. Given that Cluster 0 denotes airway epithelial cells, Extended Data Fig. 7d), the presence of *sgPax8* in this cluster aligns with Pax8's known role in epithelial lineages. Interestingly, within C1 we observe a spatial divergence, with the cluster appearing in both upper and lower regions of our spatial map (Fig. 5c). This separation, despite the shared transcriptional identity influenced by Pax8, highlights the potential influence of different local microenvironmental cues or spatial contexts on cellular behavior, even within a defined transcriptional state, made clear by Perturb-DBiT. To further examine whether the distinct spatial patterns of sgRNAs observed in C1 and C2 tumors correlate with complex intercellular communication within the TME, we conducted a spatial ligand-receptor (L-R) analysis using the co-profiled transcriptomic data. Differential L-R analysis across all identified spatial populations revealed unique interaction profiles (Fig. 5g). Comparing C2 to C1, the top enriched L-R pairs included *Lamc1-Itgb1*, *Calm1-Cacna1c*, *Lamc1-Itga6*, and *Ntn1-Unc5b*. These signaling pathways are known to enhance extracellular matrix (ECM) organization^81^, calcium signaling^82^, tumor cell adhesion to the ECM^83^, and angiogenesis^84^, respectively. These processes may drive the divergence of C2 tumors from C1, as highlighted by the spatial perturbation analysis performed by Perturb-DBiT.

Then, to validate the immune infiltration and exclusion in sgRNA-perturbed tumors, we utilized co-detection by indexing (CODEX) with a 27-plex protein panel, including both immune and tumor cell markers (Supplementary Table 2), on an adjacent section. Herein, we focus on three anatomically identifiable tumors 1, 2, and 3. The CODEX imaging revealed significant overlap with transcriptomic cluster 4 in the lymph nodes, characterized by elevated expression of pan-B cells (Fig. 5h). Additionally, the analysis highlighted a presence of CD8^+^ T cells near the lymph nodes of the C1 tumor, potentially due to the pro-inflammatory environment facilitated by sg*Zc3h12a*-specific sgRNA enrichment. In contrast, while PD-L1 expression was nearly undetectable in C1, it was significantly elevated in C2 (Fig. 5h), indicating a more pronounced immunosuppressive tumor microenvironment.

CODEX segmentation and clustering has identified distinct cellular clusters, such as pan-B cells (cluster 3), pan-T cells (cluster 4) (Fig. 5I and Fig. S7F), yet they were limited within the tumor lesions (Fig. 5b). Nonetheless, we discerned three major CODEX clusters (Fig. 5i,j) that co-localize with GFP tumor cells: cluster 0 (CC0), cluster 1 (CC1), and cluster 2 (CC2). Notably, CC1 predominates in C1 tumors, whereas CC0 is scarce; by contrast, C2 features a disproportionately high percentage of CC0. Specifically, compared to CC1, CC0 exhibited expressed elevation of F4–80, Cd45, Cd169 and Cd11b, indicative of macrophages, alongside Cd11c and MHC II, markers characteristic of myeloid compartment dendritic cells (DCs) (Fig. 5i). The high amounts of F4–80 and Cd11b in C2 (Tumor 3) further support a significant enrichment of macrophages within its microenvironment, contrasting with the CC1-dominant C1 tumors. This disparity in myeloid cell populations suggests distinct immune landscapes between these tumor types, potentially influencing tumor progression and overall microenvironmental interactions.

Taken together, the results highlight that Perturb-DBiT is effective in capturing sgRNAs from immune-competent syngeneic mouse model bearing a genome-scale CRISPR library and concurrently sequencing spatial transcriptomics, showcasing its unparalleled capabilities in revealing tumor and tumor microenvironment heterogeneity. Specifically, in the C2 tumor, multiple tumor-related gene perturbations were detected and potentially result in an enrichment of genotypically diverse cell types and cell-cell communication patterns. These are primarily involved in modulating the tumor architecture to promote tumor progression or migration. Moreover, DCs and TAMs may engage in tumor-supporting procedures within the immunosuppressive TME, contributing to the heightened malignancy of the tumors.

## Discussion

In this study, we introduced Perturb-DBiT, a cutting-edge technology seamlessly integrating spatially resolved detection of sgRNA via base-by-base sequencing with total RNA spatial transcriptome profiling on the same tissue section for spatially resolved in vivo CRISPR screening, offering profound insights into the functional landscape of complex in vivo biological systems. By enabling the simultaneous profiling of genetic perturbations and total RNA transcriptomes across various experimental *in vivo* models, Perturb-DBiT enhances our understanding of how genetic modifications influence biomolecular regulation and cellular behaviors within the tissue microenvironment. This versatile platform employs two robust approaches, Perturb-DBiT PAC and DC, to accomplish co-profiling of the biomolecules of interest, accommodating a range of delivery vectors and sgRNA library sizes, and is compatible with both FFPE and fresh frozen samples, making it adaptable for diverse experimental contexts.

This technology enables the unbiased spatial uncovering of perturbation-associated changes in total RNA transcriptomes and post-transcriptional regulation and is compatible with CRISPR libraries of any size through its base-by-base sgRNA sequencing capability. Specifically, Perturb-DBiT enables the spatial profiling of non-coding RNAs, marking the first instance of such detection within a spatial CRISPR screen. This capability expands functional insights that can be derived from spatial perturbation experiments, as non-coding RNAs play crucial roles in gene regulation, chromatin remodeling and cellular response. By capturing their spatial distribution alongside the co-profiling of both sgRNAs and mRNAs, Perturb-DBiT provides a more comprehensive view of gene regulatory networks within the tissue microenvironment, potentially uncovering novel non-coding RNA-mediated mechanisms that contribute to tumor progression, metastasis, and immune modulation. Further, we have shown that Perturb-DBiT elucidates tumor suppressor and promoter programs, validated by in vitro data. This spatial resolution empowers the discovery of novel molecular pathways and interactions that drive tumor initiation and progression and tumor clonal dynamics, enhancing our understanding of how genetic modifications influence the spatial organization and immune composition within the TME. This comprehensive approach not only broadens the scope of genetic inquiry, but also lays the groundwork for developing targeted therapeutic strategies based on spatial dynamics. These dynamics, identified by Perturb-DBiT as pivotal in shaping the tumor architecture and immune composition of the TME, hold potential for developing innovative immunotherapies in the future.

However, Perturb-DBiT has yet to demonstrate genome-wide CRISPR screening, primarily due to limited sequencing depth and restricted coverage of tissue areas. We anticipate that sequencing tens of serial sections from an entire tumor could capture thousands of unique sgRNAs. Nonetheless, our analysis of five consecutive tissue sections revealed that approximately half of the top-ranked sgRNAs were consistently detected across these sections. This finding highlights the ability to identify the most relevant perturbations, even in the absence of comprehensive genome-wide screening. Another limitation of our current method is yet to realize true single-cell resolution, which may be achieved by fully integrating sequencing-based and imaging-based spatial omics approaches or by further reducing the spot size^85^–^88^. To combine the strengths of histological imaging and deep learning, we applied iStar to produce super-resolved single-cell transcriptome data (fig. S3), representing a promising avenue towards this goal. Even with these limitations, this technology provides critical spatial information about sgRNA distribution in an unbiased and base-by-base sequencing fashion which is compatible with using a genome-scale sgRNA library and demonstrated consistent capture of biological relevant sgRNA hits across multiple tissue sections in our studies.

## Methods

### Mouse Models

For SMARTA TCR transgenic and B16-OVA tumor models, mice were housed and bred at the St. Jude Children's Research Hospital Animal Resource Center in specific pathogen-free conditions. C57BL/6, *Rosa26-Cas9*^41^ and SMARTA TCR transgenic^89^ mice were purchased from the Jackson Laboratory. Female and male mice were used at 6–10 weeks of age. Mice were housed and bred at the St. Jude Children's Research Hospital Animal Resource Center in specific pathogen-free conditions. The research conducted in this study complied with all the relevant ethical regulations. Experiments and procedures were approved by and performed in accordance with the Institutional Animal Care and Use Committee (IACUC) of St. Jude Children's Research Hospital. *Rosa26-Cas9* transgenic mice were injected subcutaneously with 1 × 10^6^ B16-OVA-Cas9 cells expressing the CRISPR sgRNA library in the right flank. Tumor tissues were harvested 14 days after implantation. Tumor size limits were approved to reach a maximum of 3,000 mm^3^ or ≤ 20% of body weight (whichever was lower) by the IACUC of St. Jude Children's Research Hospital.

For CRISPR GEMM model of liver cancer: mixed gender (randomized males and females) 8–12 week old Rosa26-LSL-Cas9-2A-EGFP (LSL-Cas9) mice were bred with C57BL/6J mice, FVB.129S6(B6)-Gt(ROSA)26Sortm1(Luc)Kael/J mice, or C57BL/6N-Gt(ROSA)26Sortm13(CAG-MYC,-CD2*)Rsky/J mice^42^.

For HT29 lung metastatic colonization model: 8-week-old NSG mice were used. For E0771 syngeneic lung tumors: 8-week-old female C57BL/6J mice were used. This study operated under institutional regulatory approval. All work was performed under the guidelines of Yale Environment, Health and Safety (EHS) Committee with an approved protocol. Animal work for CRISPR GEMM model of liver cancer, HT29 lung metastatic colonization model and E0771 syngeneic lung tumors operated under the guidelines of Yale University Institutional Animal Care and Use Committee (IACUC) with approved protocols (Chen 20068). Please refer to previously published work for method details^42^.

### Sample handling and preparation of tissue sections

Fresh tissue sections were obtained from the mentioned mouse specimens. These sections were preserved in PFA and subsequently prepared for paraffin embedding. To maintain RNA, DNA, and protein integrity, all procedures from tissue collection to embedding were carried out in a contamination-free environment. The stained tissue sections were then examined by experienced histologists to confirm their suitability for further analysis.

Paraffin blocks from and mouse samples were sliced into 7–10 micrometer-thick sections. These sections were then placed on glass slides coated with Poly-L-Lysine. Additional serial sections were collected simultaneously for Perturb-DBiT. While the sectioning of mouse formalin-fixed paraffin-embedded (FFPE) samples was performed at the YPTS facility, the sectioning of mouse frozen (FF) samples was carried out in-house. The prepared paraffin sections were subsequently stored at a temperature of −80 degrees Celsius until required for further analysis.

### B16-OVA tumor model: cell lines and retroviral library transduction

B16-OVA cell line was provided by D. Vignali (University of Pittsburgh). The HEK293T cell line was purchased from the American Type Culture Collection (ATCC). The Plat-E cell line was provided by Y.-C. Liu (La Jolla Institute of Immunology). All cell lines were cultured in Dulbecco's modified essential medium (DMEM) (Gibco) or RPMI-1640 medium (Gibco) supplemented with 10% (*v*/*v*) FBS and 1% (*v*/*v*) penicillin-streptomycin at 37°C with 5% CO_2_. No commonly misidentified cell lines (International Cell Line Authentication Committee) were used in this study. Cell lines were tested and determined to be free of mycoplasma contamination. The aforementioned cell lines were not independently authenticated. To generate Cas9-expressing tumor cell line B16-OVA-Cas9, lentivirus was produced by co-transfecting Lenti-Cas9-GFP (86145, Addgene) plasmid with psPAX2 (12260, Addgene) and pMD2.G (12259, Addgene) packing plasmids into HEK293T cells. The supernatant containing viral particles was harvested 48 hours after transfection. B16-OVA cells were transduced with viral supernatant for 48 hours in RPMI-1640 (for B16-OVA) + 10% (*v*/*v*) FBS supplemented with 10 μg/ml polybrene (Sigma), followed by sorting of transduced (GFP^+^) into single clones, followed by expansion. Cas9 expression was verified by immunoblot analysis^90^. To generate tumor cells transduced with the retroviral CRISPR sgRNA library (LMA-DC-EFS vectors used as backbone) targeting epigenetic genes, retrovirus was produced by co-transfecting the retroviral library with pCL-Eco (12371, Addgene) and VSV.G (14888, Addgene) packing plasmids into Plat-E cells. The supernatant containing viral particles was harvested 48 hours after transfection. B16-OVA-Cas9 cells were transduced with viral supernatant for 48 hours in RPMI 1640 (for B16-OVA) + 10% (*v*/*v*) FBS supplemented with 10 μg/ml polybrene (Sigma), followed by sorting Ametrine+ cells. Cells were cultured for another 14 days for genome editing and expansion.

### B16-OVA tumor model: Dual-guide direct-capture retroviral library construction

For the curated gene list containing 38 epigenetic genes, a total of two gRNA sequences distributed on an individual construct were designed for each gene. To construct the library, a customized oligonucleotide pool containing 43 oligonucleotides targeting those 38 epigenetic genes and 10 NTCs (each oligonucleotide contains two guides targeting the same gene or NTC) (Supplementary table 4) was ordered from Twist Biosciences. The oligonucleotide design follows the overall structure: 5′-PCR adapter-CCACCTTGTTGG-protospacer A–GTTTCAGAGCAGTCTTCGTTTTCGGGGAAGACAAGAAACATGG-protospacer B–GTTTAAGAGCTAAGC-PCR adapter-3′. The dual-guide library was generated using a two-step cloning strategy as previously described^91^. In brief, the PCR-amplified oligonucleotide pool was digested with BstXI and Bpu1102I (Thermo Fisher) and ligated into a similarly digested LMA-DC-EFS vector. The ligation product was then electroporated into Endura Duos (Lucigen) and amplified, and the resulting intermediate library was assessed for quality using next generation sequencing (NGS). For quality control, sgRNA skewing was measured using the script calc_auc_v1.1.py^92^ to monitor how closely sgRNAs are represented in a library, and sgRNA distribution was plotted with the area under the curve < 0.7 to pass quality control. The Python script count_spacers.py^93^ was used as an additional measure for quality control. Next, the CR3^cs1^-hU6 insert from pJR89 (140096, Addgene) was isolated by digestion with BsmBI followed by gel extraction. The intermediate library from above was digested with BbsI and treated with rSAP. Finally, the CR3^cs1^-hU6 insert was ligated into the intermediate library vector, purified by isopropanol purification and electroporated into Endura Duos. Electroporated cells were plated overnight at 32°C, collected the next day and the plasmid library extracted using endotoxin-free maxiprep kits (Qiagen). The amplified library was then validated by NGS as described above.

#### SMARTA TCR transgenic model: sgRNA transduction in CD4 ^+^ T cells and adoptive transfer after LCMV infection

Naive Cas9-expressing SMARTA cells were isolated from the spleen and peripheral lymph nodes of Cas9-SMARTA mice using the naive CD4^+^ T cell isolation kit according to the manufacturer's instructions. Purified naive SMARTA cells were activated *in vitro* for 18 h with 5 μg/ml anti-CD3 and 5 μg/ml anti-CD28 before viral transduction. Viral transduction was performed by spin-infection at 800*g* at 25°C for 3 h with 10 μg/ml polybrene (Sigma). Cells were then cultured with human IL-2 or mouse IL-7 for 4 days. Transduced cells were sorted based on the expression of Ametrine using a Reflection (i-Cyt) and then adoptively transferred into C57BL/6 recipients 1 day after infection of the host mice with LCMV. For LCMV infection, 2 × 10^5^ plaque-forming units (PFU) of the Armstrong strain of LCMV were injected intraperitoneally. sgRNAs were designed using an online tool (https://portals.broadinstitute.org/gpp/public/analysis-tools/sgrna-design). sgRNAs used in this study were cloned into LMA-DC-EFS dual-guide direct-capture retroviral sgRNA vectors described before^37^. The spacer sequences for the two non-targeting control sgRNAs on the dual-guide retroviral vector: 5′-AGGACTATCCGCGGGATTAG - 3′ and 5′- ATGACACTTACGGTACTCGT - 3′; At day 6 post LCMV infection, spleens were harvested for section.

### Lentivirus production for HT29 lung metastatic colonization model and E0771 syngeneic model:

For lentivirus production, 20 μg of lentiviral vectors—such as Lenti-EF1a-GFP-T2A-Luciferase-WPRE, Lenti-EF1a-Cas9-Blasticidin-WPRE, and either a target sgRNA or library in Lenti-U6-sgRNA-EFS-Puro-WPRE, were co-transfected with 10 μg of pMD2.G and 15 μg of psPAX2 into LentiX293T cells. This co-transfection was carried out in a 150 mm dish at 80–90% confluency using 135 μL of lipoD293 transfection reagent. Virus-containing supernatant was harvested at 48 hours post-transfection, then centrifuged at 1,500 g for 15min to remove cellular debris. The virus was subsequently concentrated using a LentiX concentrator, aliquoted, and stored at −80°C. The library virus was titrated by infecting HT29 or E0771 cells and applying a selection pressure of 3 μg/ml puromycin.

### Generation of human Brunello library or mouse Brie library in transduced cells:

HT29 or E0771 cells stably expressing GFP and luciferase were created by transducing them with lentivirus EF1a-GFP-T2A-Luciferase-WPRE. Cells expressing GFP were isolated using flow cytometry sorting. Subsequently, HT29-GFP-Luciferase or E0771-GFP-Luciferase cells stably expressing Cas9 were produced by transduction with lentivirus EF1a-Cas9-Blasticidin-WPRE, followed by a selection period of 7 days using 20 μg/ml blasticidin. The Brunello library (Supplementary Table 4) was transduced into HT29-Cas9 cells, while the Brie library (Supplementary Table 4) was used to infect E0771-Cas9 cells. The sgRNA libraries were transduced at a calculated MOI of 0.3, with a minimal representation of over 200x transduced cells per sgRNA. Post-infection, cells were incubated at 37°C for 24 hours before selection in media containing 3 μg/ml puromycin for 3 days. The successfully transduced cells were then injected into mice.

### HT29 lung metastatic colonization model:

1 × 10^^6^ HT29-Cas9-Brunello library cells were intravenously injected into 8-week-old NSG mice. The formation of metastases was monitored using IVIS imaging.

### E0771 syngeneic lung colonization model:

1 × 10^^6^ E0771-Cas9-Brie library cells were intravenously injected into 8-week-old female C57BL/6J mice. The formation of metastases was also monitored using IVIS imaging.

### Clonogenic assay of HT29 cells in vitro:

HT29-Cas9 cells were transduced with either a non-targeting control (NTC), AAVS1 sgRNA control, or target gRNA lentivirus (Lenti-EF1a-gRNA-EF1a-Puro). The gRNA-transduced cells underwent selection via puromycin for 3 days, followed by validation of sgRNA genome editing using the T7E1 assay. For the tumor cell clonogenic assay, cells were dissociated into a single-cell suspension. This suspension was diluted and seeded at densities of 5,000 cells per well in a 6-well plate. After 14 days, the medium was removed, and the cells were gently rinsed with PBS, fixed with 75% ethanol, and stained with 0.5% crystal violet. Cell colonies were analyzed using the Colony Area plugin in ImageJ, which automatically quantifies colony formation in clonogenic assays^94^. Gene oligos used for assay are listed in table (Supplementary Table 5)

### Microfluidic Device Fabrication for Perturb-DBiT

The microfluidic device was fabricated using a standard soft lithography process, detailed in our previous publication^34^. In summary, customized high-resolution photomasks were printed and procured from Front Range Photomasks (Lake Havasu City, AZ). Photomasks were then cleaned with acetone to remove contaminants and master wafers were created using SU-8 negative photoresist (SU-2010 or SU-2025) on silicon wafers. Fabrication widths varied between 10, 20, and 50 microns. The wafers were treated for 20 minutes with chlorotrimethylsilane to achieve high-fidelity hydrophobic surface. Polydimethylsiloxane (PDMS) microfluidic chips were produced using a 10:1 mixture of base and curing reagents (following the manufacturer's protocol) and poured over the master wafers. The mixture was degassed in a vacuum for 30 minutes and subsequently cured at 70°C for a minimum of 2 hours. Upon solidification, the PDMS devices were cut out and inlets and outlets were punched prior to Perturb-DBiT application.

### DNA barcodes annealing

The DNA oligos used in this study were sourced from Integrated DNA Technologies (IDT, Coralville, IA) with the sequences provided in Supplementary Table 3. Barcode (100 μM) and ligation linker (100 μM) were mixed in equal parts (1:1) in a 2X annealing buffer (20 mM Tris-HCl pH 8.0, 100 mM NaCl, 2 mM EDTA). The annealing process followed this thermal cycling protocol: 95°C for 5 min, gradual cooling to 20°C at a rate of −0.1°C/s, and then a 3-minute hold at 12°C. The annealed barcodes can be preserved at −20°C until use.

### FFPE tissue deparaffinization and decrosslinking

A tissue section was retrieved from −80°C storage and allowed to sit at room temperature for 10 minutes until moisture had evaporated. The tissue slide was then baked at 1–2 hours at 60°C to soften and melt the paraffin. Paraffin was removed by immersing the slides into two changes of xylene, followed by rehydration through a graded series of ethanol solutions for five minutes each, including two rounds of 100% ethanol and one round each of 90%,70% and 50% ethanol, with a final rinse of distilled water. The slide was then completely covered by a pre-boiled 1X antigen retrieval buffer (on a hot plate) for 10 minutes followed by a 15-minute cooldown to room-temperature on ice. After a brief rinse in distilled water, the tissue was scanned using a 10x objective on the EVOS M7000 Imaging System to capture the intact tissue. Sections could either be used right away for Perturb-DBiT, or could be stored for up to 4-weeks at −80°C.

### Perturb-DBiT DC permeabilization, reverse transcription, and spatial barcoding with microfluidic devices

The tissue was permeabilized at room temperature for 20 min with 1% Triton X-100 in DPBS, followed by a 0.5X DPBS-RI (1X DPBS diluted with nuclease-free water, 0.05 U/μL RNase Inhibitor) wash to stop permeabilization. The tissue slide was then air-dried, imaged, and equipped with a PDMS reservoir for bulk reverse transcription. 62.2uL of the reverse transcription mix, using a 1:1 ratio of sgRNA: mRNA capture reagents (10 μL 25 μM Transcriptome RT Primer, 10 μL 25 μM CRISPR RT Primer, 20 μL 0.5X DPBS-RI, 12 μL 5X RT Buffer, 6 μL 200U/μL Maxima H Minus Reverse Transcriptase, 3 μL 10mM dNTPs, 0.8 μL 20 U/μL SUPERase•In RNase Inhibitor, 0.4 μL 40 U/μL RNase Inhibitor) was added into the PDMS reservoir and sealed with parafilm prior to incubation in a wet box for 30 min at room temperature. The sample was then incubated for 90 min at 42°C, followed by a 5 min 50mL DPBS shake-wash.

To ligate barcode A and C *in situ*, the first PDMS device was positioned on top of the tissue slide, ensuring alignment of the center channels with the ROI. The device was then imaged to record its positioning on the tissue for downstream analysis and alignment. Next, an acrylic clamp was applied to firmly secure the PDMS device to the tissue slide, preventing reagents from leaking between the microfluidic channels. The ligation mix (100 μL 1X NEBuffer 3.1, 11.3 μL nuclease-free water, 26 μL 10X T4 ligase buffer, 15 μL T4 DNA ligase, 5 μL 5% Triton X-100, 2 μL 40 U/μL RNase Inhibitor, and 0.7 μL 20 U/μL SUPERase•In RNase Inhibitor) was then prepared on ice. For the barcoding reaction, a total of 5 μL ligation solution (3 μL ligation mix and 1 μL each of barcode A and C) was carefully pipetted into each of the 50 inlets of the device at room temperature. The ligation solution within each of the 50 inlets was then gently pulled throughout each of the 50 channels of the device using a delicately adjusted vacuum. After incubation in a wet box for 30 min at 37°C, the first PDMS device was removed, and the slide was washed with 50mL DPBS. The second PDMS device, featuring 50 channels perpendicular in direction to the first PDMS device, was then attached to the tissue slide with careful alignment of the central channels as-to finalize the positioning of the ROI. Following another microscopic image for downstream alignment, the acrylic clamp was applied once again to firmly secure the PDMS device in place and the ligation of barcode B and D was similarly performed. Following barcoding, five flow-washes with 500 μL nuclease-free water were performed and the final scan was conducted to record the tissue-imprinted microchannel marks, making clear the ROI.

#### Perturb-DBiT PAC permeabilization, in situ polyadenylation, reverse transcription, and spatial barcoding with microfluidic devices

The tissue was permeabilized and washed as described above. The tissue was then air-dried and equipped with a PDMS reservoir surrounding the ROI. *In Situ* polyadenylation was performed using *E.Coli* Poly(A) Polymerase. Samples were first equilibrated incubating 100 μL poly(A) wash buffer (88 μL nuclease-free water, 10 μL 10X Poly(A) Reaction Buffer, 2 μL 40 U/μL RNase Inhibitor) at room temperature for 5 min. After removing the Poly(A) wash buffer, 60 μL of the Poly(A) enzymatic mix (38.4 μL nuclease-free water, 6 μL 10X Poly(A) Reaction Buffer, 6 μL 5U/μL Poly(A) Polymerase, 6 μL 10mM ATP, 2.4 μL 20 U/μL SUPERase•In RNase Inhibitor, 1.2 μL 40 U/μL RNase Inhibitor) was added to the reservoir and incubated in a wet box at 37°C for 25 minutes. Tissue slides were then shake-washed in DPBS as described above before adding 60.2uL of the reverse transcription mix(10 μL 25 μM Transcriptome RT Primer, 20 μL 0.5X DPBS-RI, 12 μL 5X RT Buffer, 6 μL 200U/μL Maxima H Minus Reverse Transcriptase, 8 μL nuclease-free water, 3 μL 10mM dNTPs, 0.8 μL 20 U/μL SUPERase•In RNase Inhibitor, 0.4 μL 40 U/μL RNase Inhibitor) to the PDMS reservoir with a similar incubation as described above. To ligate barcode A *in situ*, the first PDMS device was positioned and imaged as previously described. The ligation mix (100 μL 1X NEBuffer 3.1, 61.3 μL nuclease-free water, 26 μL 10X T4 ligase buffer, 15 μL T4 DNA ligase, 5 μL 5% Triton X-100, 2 μL 40 U/μL RNase Inhibitor, and 0.7 μL 20 U/μL SUPERase•In RNase Inhibitor) was then prepared on ice. For the barcoding reaction, a total of 5 μL ligation solution (4 μL ligation mix and 1 μL of barcode A) was carefully pipetted into each of the 50 or 100 inlets of the device at room temperature. The ligation solution within each of the 50 or 100 inlets was then gently pulled throughout each of the 50 or 100 channels of the device using a delicately adjusted vacuum. After incubation in a wet box for 35 min at 37°C, the first PDMS device was removed, and the slide was washed as described above. The second PDMS device, featuring 50 or 100 channels perpendicular in direction to the first PDMS device, was then similarly attached and imaged. The ligation of barcode B was similarly performed. Following barcoding, five flow-washes with 500 μL nuclease-free water were performed and the final scan was conducted to record the tissue-imprinted microchannel marks, making clear the ROI.

### Tissue lysis and extraction of cDNA

For both Perturb-DBiT DC and PAC, the ROI of the barcoded tissue was surrounded with a clean PDMS reservoir and clamped securely with an acrylic chip. A 2X lysis buffer (20 mM Tris-HCl pH 8.0, 400 mM NaCl, 100 mM EDTA, and 4.4% SDS) was prepared ahead of time. 110 μL of lysis mix (50 μL 1X DPBS, 50 μL 2X lysis buffer, 10 μL 20 μg/μL Proteinase K solution) was then added to the reservoir and sealed with parafilm prior to a 2 hour incubation at 55°C in a wet box. Following the reaction, 110 μL sgRNA and mRNA cDNA-rich liquid was collected into a 1.5mL DNA low-bind tube. The tissue lysate could then be stored at −80°C prior to the next steps.

### Purification of cDNA and template switching

The 100 μL sgRNA and mRNA cDNA was purified using 40 μL of Dynabeads MyOne Streptavidin C1 beads resuspended in 100 μL of 2X B&W buffer (10 mM Tris-HCl pH 7.5, 1 mM EDTA, 2 M NaCl) and incubated with rotation at room temperature for 60 min to ensure sufficient binding. Following magnetic separation and two washes with 1X B&W buffer (with 0.05% Tween-20) and an additional two washes with 10 mM Tris-HCl pH 7.5 containing 0.1% Tween-20, cDNA molecules bound with streptavidin beads were then resuspended in 200 μL of TSO Mix (75 μL nuclease-free water, 40 μL 5X RT buffer, 40 μL 20% Ficoll PM-400, 20 μL 10mM dNTPs, 10 μL 200U/μL Maxima H Minus Reverse Transcriptase, 5 μL 40 U/μL RNase Inhibitor, 10 μL 100 μM TSO Primer). Template switching was performed with rotation at room temperature for 30 min and then at 42°C for another 90 min. Beads then went through two washes, one with 10mM Tris-HCl pH 7.5 containing 0.1% Tween-20 and another with nuclease-free water. The beads were then PCR amplified according to different protocols, depending on if Perturb-DBiT DC or PAC was employed.

### Perturb-DBiT DC PCR amplification and library preparation

Washed beads were then resuspended in 200 μL of PCR mix with a 1:1 ratio of sgRNA: mRNA capture primers (100 μL 2X KAPA HiFi HotStart ReadyMix, 76 μL nuclease-free water, 8 μL 10 μM PCR Primer 1, 8 μL 10 μM PCR Primer 2, 8 μL 10 μM PCR Primer 3). This mixture was distributed into 4 PCR strip tubes and the following PCR program was used: 95°C for 3 min, with five cycles at 98°C for 20 s, 63°C for 45 s, 72°C for 3 min, followed by an extension at 72°C for 3 min and 4°C hold. After magnetically removing beads, 19 μL of the PCR solution was combined with 1 μL 20X EvaGreen for quantitative real-tiyme PCR (qPCR) using the same program while the remaining solution underwent normal PCR simultaneously. The cycle numbers were finally determined when ½ of the saturated signal was observed. The full PCR product was then purified using a 0.6X ratio of SPRIselect beads while saving the supernatant. The pellet portion (transcriptomic cDNA) adhered to the standard manufacturer's instructions for purification while 150 μL supernatant (sgRNA cDNA) underwent a second 1.2X SPRI selection. Following a second elution in 50 μL nuclease-free water, a 1.0X Spri selection was performed with the transferred supernatant. Finally, sgRNA product was eluted in 30 μL nuclease-free water. The transcriptomic cDNA and sgRNA each underwent analysis using a TapeStation system with D5000 DNA reagents and ScreenTape. Depending on experiment-specific sgRNA cDNA QC, sgRNA enrichment was necessary. We prepared a sgRNA enrichment PCR mix (50 μL 2X KAPA HiFi HotStart ReadyMix, 2ng sgRNA product from the previous reaction, 8 μL 10 μM PCR Primer 2, 8 μL 10 μM CRISPR PCR Primer 3, 5uL 20X EvaGreen nuclease-free water to 100uL) using a 100uL system and performed qPCR using the same program. After enrichment, sgRNA underwent a 1.2X SPRI cleanup prior to elution in 30 uL nuclease-free water. After ensuring high-quality cDNA, we performed direct ligation library preparation for sequencing. We prepared two solutions, each consisting of 50 μL 2X KAPA HiFi HotStart ReadyMix, ~2ng sgRNA or mRNA cDNA, 4 μL 10 μM P5 Primer, and 4 μL 10 μM P7 Primer or CRISPR P7 Primer for transcriptome or sgRNA library, respectively. We then performed qPCR for each sample using the aforementioned program to build libraries. The transcriptome library then underwent purification using a 0.8X SPRI ratio, while the sgRNA library then underwent purification using a 1.2X SPRI ratio. Both libraries were quality control checked using TapeStation and then were sequenced on an Illumina NovaSeq 6000 Sequencing System with a paired-end 150bp read length.

### Perturb-DBiT PAC PCR amplification, rRNA removal, and library preparation

For the Perturb-DBiT PAC method, washed beads were then resuspended in 200 μL of PCR mix (100 μL 2X KAPA HiFi HotStart ReadyMix, 84 μL nuclease-free water, 8 μL 10 μM PCR Primer 1, and 8 μL 10 μM PCR Primer 2). This mixture was distributed into 4 PCR strip tubes and the following PCR program was used: 95°C for 3 min, with 13 cycles at 98°C for 20 s, 63°C for 45 s, 72°C for 3 min, followed by an extension at 72°C for 3 min and 4°C hold. The full PCR product was then purified using a 0.7X ratio of SPRIselect beads while saving the supernatant. The pellet portion (transcriptomic cDNA) adhered to the standard manufacturer's instructions for purification and was eluted in 20 μL nuclease-free water while the transferred supernatant (containing sgRNA) underwent a second 1.2X SPRI selection before being eluted in 20 μL nuclease-free water.

The resulting mRNA cDNA and sgRNA underwent analysis using a TapeStation system with D5000 DNA reagents and ScreenTape. The SEQuoia Ribodepletion Kit was then used to eliminate rRNA-derived fragments and mitochondrial rRNA from the amplified sgRNA and cDNA product, following manufacturer's guidelines. Based on the readout from TapeStation analysis, 20ng of sgRNA and mRNA cDNA was used as an input amount and two rounds of rRNA depletion were performed. Next, the aforementioned PCR program was executed on the two separate samples for 7 cycles to directly ligate sequencing primers using a 100 μL system (50 μL 2X KAPA HiFi HotStart ReadyMix, ~ 42 μL solution from the rRNA removal step, 4 μL 10 μM P5 Primer, and 4 μL 10 μM P7 Primer). The final transcriptomic library underwent 0.7X SPRI selection, while the final sgRNA library underwent 0.6X SPRI selection followed by a 1.2X SPRI selection. The two libraries were checked for quality control using TapeStation and then were sequenced on an Illumina NovaSeq 6000 Sequencing System with a paired-end 150bp read length.

### CODEX spatial phenotyping using PhenoCycler-Fusion

The CODEX PhenoCycler-Fusion protocol (Link to protocol) for fresh frozen tissue sections from Akoya Biosciences was followed. The tissue slide was removed from the − 80C freezer and placed on drierite beads for 5 minutes. After this, the tissue slide was immersed in acetone for 10 mins and then allowed to dry for 2 minutes. The tissue slide was then passed through a series of hydration steps – immersed in hydration buffer for 2 minutes twice, followed by fixation for 10 minutes using 1.6% PFA. Finally, the tissue section was immersed in staining buffer for 30 minutes and the antibody cocktail prepared. The tissue slide was incubated with the antibody cocktail at room temperature for 3 hours in a humidity chamber. After incubation, the tissue underwent a series of steps including post-fixation, ice-cold methanol incubation, and a final fixation step. Attached to the flow cell, the tissue section was incubated in 1X PhenoCycler buffer with additive for at least 10 minutes to improve adhesion. The CODEX cycles were then set up, the reporter plate was prepared and loaded, and the imaging process began. A final qptiff file was generated, at the end which could be viewed using QuPath V0.5^95^. Specific details regarding the PhenoCycler reagents, volumes, panels, and cycles can be found in Supplementary Table 2.

### Immunofluorescent staining

Immunofluorescence analysis of spleen tissue sections was performed following rehydration in PBS, and subsequent blocking of non-specific binding using blocking buffer comprised of PBS containing 1% BSA, 0.05% Tween-20 and 5% normal donkey serum. Slides were incubated overnight at 4°C in PBS containing 1% BSA and AF488-labeled B220, AF647-labeled CD3. Slides were washed in PBS and mounted in Vectashield vibrance mounting media containing DAPI (Vector labs, H-1800). Images were acquired an A1RHD resonant scanning confocal comprised of a Ti2-E inverted microscope equipped with an A1RHD25 scanner detector, Plan Apo 20X 1.0 NA objective, laser lines as appropriate for 405/488/647 illumination and detection and were analyzed using NIS-Elements software (Nikon Instruments; version 5.42.04).

### Wound healing assay

#### Cell Culture and Seeding:

HT-29 cells were maintained in complete growth medium supplemented with 10% fetal bovine serum (FBS). Cells were subcultured by removing the spent medium, washing with calcium- and magnesium-free PBS, and incubating with Accutase (0.5 mL for 6-well plates) for 8–10 minutes at room temperature. Detached cells were resuspended in fresh medium, centrifuged at 300×g for 3 minutes, and reseeded at a density of 3 × 10^4^ cells/cm^2^. For a 6-well plate, 1 × 10^5^ cells/well were seeded in 2 mL of medium. After 12 hours, the medium was refreshed, and cells were cultured until reaching ~ 90% confluence.

#### Scratch Assay and Imaging:

A uniform scratch wound was introduced using a P200 pipette tip on the confluent monolayer. Detached cells were removed by washing with PBS, and serum-free medium was added to minimize proliferation effects. Phase-contrast images of the initial wound (0h) were captured, followed by incubation at 37°C with imaging at 6h, 12h, 24h, and 48h. Wound closure was quantified using ImageJ, calculating the percentage of area closure over time.

### Sequence alignment and expression matrix generation of mRNA and non-coding RNA

The FASTQ file Read 2 underwent processing from the raw sequencing data, involving UMI and spatial barcode extraction. Read 1 containing cDNA sequences was trimmed using Cutadapt V3.4^96^ and then aligned to either the mouse GRCm38-mm10 or human GRCh38 reference genome using STAR V2.7.7a^97^. Utilizing ST_Pipeline V1.7.6^98^, spatial barcode sequences were demultiplexed based on the predefined microfluidic coordinates and ENSEMBL IDs were converted to gene names. This in turn generated the gene-by-spot expression matrix for downstream analysis. Matrix entries corresponding to spot positions with no underlying tissue were excluded for analysis and missing spots are inferred from nearby data to facilitate clustering analysis across the entire mapped area.

To generate the whole-transcriptome expression matrix, which includes most non-coding RNA species, we employed ASTRO (https://github.com/gersteinlab/ASTRO, v0.2)^99^. Raw FASTQ files were provided as input, and ASTRO outputted the feature-by-spot expression matrix. The gene annotation reference used in ASTRO was constructed by ASTRO's annotation-building pipeline, which integrates data from GENCODE (GENCODE 2025), GtRNAdb 2.0, and RNAcentral. Spots corresponding to positions without tissue were excluded from the matrix and missing spots were inferred using neighboring spot data to facilitate clustering analysis across the entire spatially mapped area.

### Gene expression analysis

Gene expression (GEX) data were loaded into a Seurat object along with spatial positions. The GEX data were normalized and scaled using SCTransform method with the “v2” strategy for variance-stabilizing transformation ^100^. The data were then dimensionally reduced by principal components analysis (PCA), followed by UMAP (min.dist = 0.01) using the PCA components that explained the majority of the data variance (chosen by elbow plot method)^14^,^101^. Spots were clustered by constructing a shared nearest-neighbors (SNN) graph from PCA data and using the FindClusters function (algorithm = 4). The optimal clustering resolution for modularity optimization was chosen based on the minimal total within-cluster sum-of-squares and corresponding maximal average silhouette-width when considering > 3 clusters.

DE between GEX spot clusters was performed by Wilcoxon rank sum test (FindAllMarkers function in Seurat package) using annotated genes. DE genes (adjusted p < 0.05, > 20% detected expression in cluster) were visualized by heap map (ComplexHeatmap R package v2.12.1) using the top 10 overexpressed genes from each GEX cluster. Pseudotime trajectory analyses were done using the standard Monocle 3 pipeline (R package v1.3.1) with data that were already normalized using Seurat ^102^.

### Comparative analysis of pooled and perturb-DBiT CRISPR screen detection

Perturb-DBiT was benchmarked against the traditional pooled CRISPR screen detection using frozen liver sections from the Wang et al. screen of liver tumorigenesis genes ^40^. Briefly, a receiver-operator-curve (ROC) was constructed using sgRNA detection in the pooled screen data (> 25% detection across tumor sample lobes) as the predictor for the perturb-DBiT sgRNA area (area > 2) as the response. The ROC and area-under-curve analyses were performed using the pROC R package v1.18.0.

### Preprocessing spatial screen data

A custom shell script was created to preprocess the screen data for perturb-DBiT using a combination of Cutadapt (v3.4) ^96^, Bowtie (v1.3.0) ^103^, and custom scripts. Briefly, a “whitelist” of possible unique molecular identifiers (UMIs) was created from the data using cutadapt with the following settings: -e 0.1 -m 10 -M 12 -l 10 --max-n 0 --discard-untrimmed. Next, alignment reference libraries were built for the UMI whitelist, as well as for the gRNA spacer sequences and the spot barcodes using Bowtie-build. All barcodes were extracted from fastq files using cutadapt using the parameters outlined in Supplementary Table 6. The Cutadapt flanking regions were also specified by the perturb-DBiT chemistry (Supplementary Table 6).

The trimmed sgRNA spacer sequences and barcodes for spatial position and UMI were then aligned to the custom libraries using Bowtie with the parameters below. Note that the parameters for mismatches and multi-matches (-v and -m, respectively), were optimized based on the reference library size and empirical testing with manually aligned reads (Supplementary Table 6f).

A position ID was created from the aligned spatial barcodes, which were then matched to corresponding the corresponding sgRNA name and UMI for each paired sequencing read ID. Lastly, the sgRNAs were quantified based on the UMI count and subsequently analyzed with the R programming language.

### Perturb-DBiT screen analysis

Screen sgRNA quantification data were added to the GEX Seurat objects. The spots were labeled by the uniquely detected sgRNA, or non-perturbed, if no sgRNAs were detected. Next, spots with multiple sgRNAs were marked as ambiguous or resolved if the top sgRNA of the spots had > = 10 counts and all other sgRNAs had 1 count. Genetic perturbation was analyzed using the Mixscape pipeline (Seurat R package v5.0.1) with slight modifications ^101^. First, perturbation signatures were calculated for all sgRNAs that were detected with an area > = 4 (all sgRNAs were used for samples with < 100 different sgRNAs), and Mixscape analysis was performed, splitting data by cluster. The Mixscape results were filtered to get a list of perturbed spots (perturbation-score > 0.2) and non-perturbed (NP) spots (perturbation-score < −0.2). All gene perturbation-scores for the filtered spots were then re-arranged into a Seurat data assay. The perturbation assay was dimensionally reduced by PCA, and the top PCs (explaining the majority of the data variance) were further reduced by UMAP. Spots were then clustered using the same strategy as described for GEX clustering. Clustered spots were labeled by the major perturbations of the cluster (perturbed gene for > 20% of cluster spots). Clusters with no major perturbations were labeled as “NP + n”, where n is the number of detected perturbations in the cluster. The perturbation clusters were characterized by the gene expression of liver tumor and metastasis-associated genes^104^–^106^. Perturbation burden was calculated as the area of spots with a detected sgRNA within a specified tumor region.

The gene expression pattern of each perturbation cluster was assessed by DE analyses using the NP perturbation cluster as the control and the same methods used for GEX DE. Pathway analyses were performed by Gene Set Enrichment Analysis (GSEA; fgsea R package v1.22.0), using biological process gene ontologies (2023 version, size < = 500 genes) ^107^, log2 fold-changes as the GSEA input, DE genes (adjusted p < 0.2), and the following parameters: function = fgseaMultilevel, gseaParam = 0.1, minSize = 2, and maxSize = 500. Lastly, the results were filtered to remove highly ambiguous pathways: “Translation”, “Transcription”, and “Gene Expression”.

### High-resolution tissue architecture analysis using iStar

The iStar algorithm is made up of three major components: a histology feature extractor, a high-resolution gene expression predictor, and a tissue architecture annotator, as detailed a previous publication^108^. H&E images were divided into smaller sections and then analyzed using a vision transformer to extract histological features. Then, a feed-forward weakly supervised trained neural network, then predicted superpixel-level gene expressions utilizing the top 1,000 most variable genes from the Perturb-DBiT expression matrix or top 16 sgRNA hits from the Perturb-DBiT sgRNA expression matrix. Clusters based on gene expression were used to segment the tissue and then were used for co-registration with the H&E-stained images. The top 10 genes associated with each cluster were identified and interpreted biologically, with input from a pathologist to refine the results based on tissue morphology.

### Co-registration and overlay between gRNA data and its histology

The brightfield image used in Perturb-DBiT was co-registered to its serial H&E-stained microscopy image via a manual control point based non-rigid co-registration workflow using a spline-based transformation model from Weave platform for Spatial Biology (Aspect Analytics NV, Genk, Belgium). The location of sgRNA measurements was precisely detected after co-registering its Barcode A and B images to the corresponding brightfield image. For this same section alignment, an automatic intensity matching co-registration workflow from Weave platform was used to find an affine transformation. After co-registering the brightfield image to its serial H&E-stained microscopy image via the manual non-rigid co-registration workflow, the sgRNA data was then precisely and directly linked to this same histology image. The pathologist annotated the H&E-stained microscopy image and placed 5 labels: 'Bronchus', 'Vessel', 'Perivascular Fať, 'Peripheral Tumor', and 'Tumor Core' via a web-based annotation tool included in the Weave platform. Finally, the sgRNA data and its serial histology data was precisely co-registered, overlaid and interactively visualized on the web-based Weave platform.

### Pseudotime analysis

Pseudotemporal analysis was performed with Monocle3^70^. Using scripts documented by a previous study^109^. Briefly, we normalized the count matrix with the preprocess_cds function (num_dim = 25). Dimensionality reduction was performed with the reduce_dimension function (reduction_method='UMAP', umap.min_dist = 0.5, preprocess_method='PCA'). Next, we used reversed graph embedding with the learn_graph function to generate a principal graph from the reduced dimension space and we used the order_cells function to order the observations in a pseudotemporal manner. Clustering analysis was performed with the cluster_cells function at a resolution of 0.1 and the graph_test function was used to identify DEGs.

### Ligand-receptor interaction analysis

Niches R package was utilized to analyze cell-cell connectivity patterns from ligand and receptor expression within our Perturb-DBiT dataset. The normalized Seurat object served as input and we applied RunNICHES using the FANTOM5 ligand-receptor database for human, specifying spatially resolved neighborhoods. Ligand-receptor interactions were inferred through NeighborhoodToCEll mode, capturing cell-specific signaling within defined spatial contexts. The output was processed for dimensionality reduction and visualization of niche-specific communication. Top-ranked interaction pairs were prioritized based on biological and statistical significance and visualized via DotPlot and spatial mapping.

### Preprocessing of CODEX data

Cell segmentation was performed using a StarDist-based model in QuPath. The DAPI channel served as the nuclear marker for cell segmentation. The mean intensity of each marker per segmented cell was exported together with the centroids of each cell as a CSV. Downstream analysis was performed using Seurat 4.3.0 package. The dataset was normalized and scaled using the NormalizeData and ScaleData functions. Linear dimensional reduction was then performed with the “RunPCA” function. The “FindNeighbors” function embedded spots into a K-nearest neighbor graph structure based on Euclidean distance in PCA space, and the “FindClusters” function was used to cluster the spots. The “RunUMAP” function was used to visually show spatial heterogeneities through the Uniform Manifold Approximation and Projection (UMAP) algorithm. The clusters were plotted spatially using the ImageDimPlot function. The FindAllMarkers function was used to find the differentially expressed proteins in each cluster and the heatmap plotted using the DoHeatMap function.

### Statistical analysis

Prism V9 (GraphPad) was used for statistical analyses and specific tests are indicated in the text.

## Data Availability

Raw and processed sequencing data for this study are in preparation for submitting to the NCBI Gene Expression Omnibus (GEO) database (accession number pending). All original code has been deposited at our GitHub space and is publicly available as of the date of publication. Any additional information required to reanalyze the data reported in this paper is available from the lead contact upon request.
